# A novel R2R3-MYB from grape hyacinth, MaMybA, which is different from MaAN2, confers intense and magenta anthocyanin pigmentation in tobacco

**DOI:** 10.1186/s12870-019-1999-0

**Published:** 2019-09-09

**Authors:** Kaili Chen, Lingjuan Du, Hongli Liu, Yali Liu

**Affiliations:** 1 College of Landscape Architecture and Arts, Northwest A&F University, Yangling, 712100 Shaanxi People’s Republic of China; 2 Key Laboratory of Horticultural Plant Biology and Germplasm Innovation in Northwest China, Ministry of Agriculture, Yangling, 712100 Shaanxi People’s Republic of China; 3 State Key Laboratory of Crop Stress Biology in Arid Areas, Northwest A&F University, Yangling, 712100 Shaanxi People’s Republic of China

**Keywords:** Anthocyanin biosynthesis, Flower color, Monocot plants, R2R3-MYB, Transcription regulation, Transgenic tobacco

## Abstract

**Background:**

The primary pigments in flowers are anthocyanins, the biosynthesis of which is mainly regulated by R2R3-MYBs. *Muscari armeniacum* is an ornamental garden plant with deep cobalt blue flowers containing delphinidin-based anthocyanins. An anthocyanin-related R2R3-MYB MaAN2 has previously been identified in *M. armeniacum* flowers; here, we also characterized a novel R2R3-MYB MaMybA, to determine its function and highlight similarities and differences between MaMybA and MaAN2.

**Results:**

In this study, a novel anthocyanin-related R2R3-MYB gene was isolated from *M. armeniacum* flowers and functionally identified. A sequence alignment showed that MaMybA contained motifs typically conserved with MaAN2 and its orthologs. However, the shared identity of the entire amino acid sequence between MaMybA and MaAN2 was 43.5%. Phylogenetic analysis showed that they were both clustered into the AN2 subgroup of the R2R3-MYB family, but not in the same branch. We also identified a IIIf bHLH protein, MabHLH1, in *M. armeniacum* flowers. A bimolecular fluorescence complementation assay showed that MabHLH1 interacted with MaMybA or MaAN2 in vivo; a dual luciferase assay indicated that MaMybA alone or in interaction with MabHLH1 could regulate the expression of *MaDFR* and *AtDFR*, but MaAN2 required MabHLH1 to do so. When overexpressing *MaMybA* in *Nicotiana tabacum* ‘NC89’, the leaves, petals, anthers, and calyx of transgenic tobacco showed intense and magenta anthocyanin pigments, whereas those of OE-*MaAN2* plants had lighter pigmentation. However, the ovary wall and seed skin of OE-*MaMybA* tobacco were barely pigmented, while those of OE-*MaAN2* tobacco were reddish-purple. Moreover, overexpressing *MaMybA* in tobacco obviously improved anthocyanin pigmentation, compared to the OE-*MaAN2* and control plants, by largely upregulating anthocyanin biosynthetic and endogenous *bHLH* genes. Notably, the increased transcription of *NtF3′5′H* in OE-*MaMybA* tobacco might lead to additional accumulation of delphinidin 3-rutinoside, which was barely detected in OE-*MaAN2* and control plants. We concluded that the high concentration of anthocyanin and the newly produced Dp3R caused the darker color of OE-*MaMybA* compared to OE-*MaAN2* tobacco.

**Conclusion:**

The newly identified R2R3-MYB transcription factor MaMybA functions in anthocyanin biosynthesis, but has some differences from MaAN2; MaMybA could also be useful in modifying flower color in ornamental plants.

**Electronic supplementary material:**

The online version of this article (10.1186/s12870-019-1999-0) contains supplementary material, which is available to authorized users.

## Background

A desirable floral color is a typical trait of ornamental plants [1]; it is mainly attributable to anthocyanins, carotenoids, and betalains [2]. Anthocyanins are the primary pigments in flowers, fruit, and other plant tissues, imparting a wide range of colors, from red to violet/blue [3]. They also function in flower pollination, enhancing the tolerance of plants to biological and abiotic stress. Moreover, they are important in the food industry, where they are used as safe and natural colorants [2]. The anthocyanin biosynthetic pathway, in which anthocyanin biosynthetic genes and their regulators are essential to producing anthocyanins, has been the subject of extensive and thorough study [4]. In recent years, genetic engineering has proven to be a promising approach to flower color modification [5]; in ornamental plants, the main approach has been molecular breeding through genetic regulation of anthocyanin synthesis [6]. Although there are many examples of successful flower modification engineering in various ornamental species, either by overexpressing or silencing of diverse anthocyanin biosynthetic genes, such as in gerbera [7], rose [4], carnation [8], and chrysanthemum [9, 10], regulatory proteins may also offer considerable potential in controlling anthocyanin biosynthesis.

Anthocyanin biosynthetic regulators include R2R3-MYB transcription factors (TFs), their partners the basic helix-loop-helix (bHLH) proteins, and WD-repeat (WDR) proteins; together, these form the MYB-bHLH-WDR (MBW) complex, in which R2R3-MYBs, which regulate anthocyanin biosynthesis, are the key TFs determining the intensity and patterning of pigmentation in plants [8]. There have been numerous studies of R2R3-MYBs in both dicot and monocot ornamental plants. In dicot ornamental plants, for example, AmRosea1, AmRosea2, and Amvenosa in *Antirrhinum majus* have been shown to control anthocyanin accumulation in different flower parts [11]. In *Lisianthus*, overexpression of *AmRosea1* leads to an increased level of anthocyanins and a changed color in the petals and sepals of transgenic plants [12]. Additionally, expression of both *AmRosea1* and *AmDelila* (which encode a bHLH protein related to anthocyanin pigmentation in snapdragon flowers) has been shown to induce a high concentration of delphinidin- (Dp-) based anthocyanins in the peel and flesh of transgenic tomato fruits [13]. GMYB10, an anthocyanin-promoting R2R3-MYB in *Gerbera hybrida*, was overexpressed in the *Gerbera* cultivar ‘Terra Regina’ (pelargonidin-based flowers), obviously enhancing the anthocyanin level, and inducing novel cyanidin (Cy) biosynthesis [7, 14]. In monocot flowers, *Lilium* LhMYB12-Lat [15], *Anthurium andraeanum* AaMYB2 [16], and *Muscari armeniacum* MaAN2 [17] have been shown to promote anthocyanin accumulation in tobacco plants. In the model plant *Arabidopsis thaliana*, PAP1 and PAP2 increased anthocyanin accumulation when overexpressing as transgenes in *Arabidopsis*, tobacco, and *Rosa hybrida* [18–20]. Therefore, identifying these anthocyanin regulators is meaningful in molecular breeding aimed at modifying flower color in ornamental plants, and will even be useful in the commercial production of anthocyanins for use as colorings in the food industry.

Grape hyacinth (*M. armeniacum*) is a species of perennial bulbous monocotyledonous plants that blossom in mid-spring, and is often used as a garden ornamental. Its flowers are a naturally deep cobalt blue color, containing Dp-based anthocyanins [21, 22], and it provides suitable material for the study of blue flower coloration in monocots [23]. In our previous study, we only screened one anthocyanin-related R2R2-MYB unigene by functional annotation in the transcriptome of *M. armeniacum* flowers [23], and then named MaAN2, which was identified that it could induce anthocyanin accumulation in tobacco, and interacted with a bHLH protein, AtTT8, to regulate the expression of anthocyanin late biosynthetic genes (LBGs; i.e., *MaDFR* and *MaANS*) [17] . In the present study, we excavated a novel anthocyanin-related R2R3-MYB TF named MaMybA, using a typical anthocyanin-regulator AtPAP1 from the *M. armeniacum* flowers transcriptome [23], by local BLASTP (BLAST protein database). We found that MaMybA differed from MaAN2, and we further verified the function of *MaMybA* in anthocyanin biosynthesis by heterologous expression in tobacco. The study aimed to determine the regulatory mechanism that governs coloration in *M. armeniacum* flowers, identify a valuable anthocyanin-promoting regulatory gene for use in the modification of flower colors in ornamental plants, and determine similarities and differences between MaMybA and MaAN2 in anthocyanin synthesis.

## Results

### Sequence alignment and phylogenetic analysis of R2R3-MYB MaMybA isolated from *M. armeniacum*

One R2R3-MYB unigene was screened with local BLASTP, using anthocyanin-related R2R3-MYB TF AtPAP1 from an *M. armeniacum* flower transcriptome [23]. Its full-length cDNA sequence was obtained by RACE-PCR. The 711 bp open reading frame (ORF) of *MaMybA* encoded 237 amino acids. The cDNA sequence was submitted to the NCBI GenBank (accession number MF663728).

**Fig. 1:**
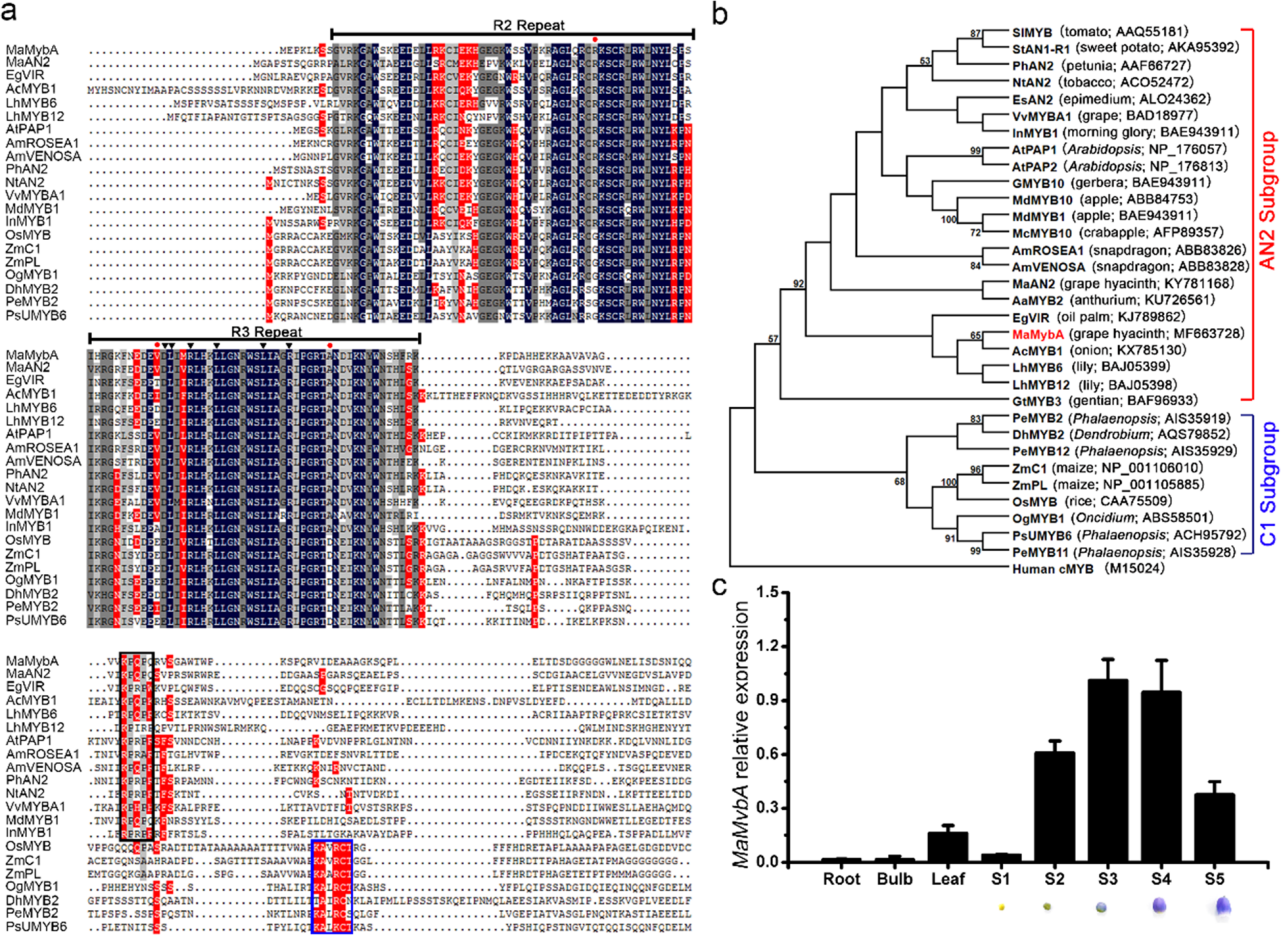
Multiple alignment, phylogenetic analysis, and transcription expression of *MaMybA*. **a** The sequence alignment of partial amino acids deduced from MaMybA, MaAN2, and other R2R3-MYBs. R2R3 repeats are indicated above the alignment. Three conserved amino acids [arginine (R), valine (V), and alanine (A)] in the R2R3 repeats of anthocyanin-promoting MYBs in dicots are marked using a red solid circle. The motif [D/E]LX_2_[K/R]X_3_LX_6_LX_3_R in the R3 repeat is necessary for interactions with a bHLH (basic Helix-Loop-Helix) protein, and is indicated by dark solid triangles. The typical motif [K/R]P[Q/R]P[Q/R] in the AN2 subgroup and KAX[K/R]C[S/T] in the C1 subgroup are shown by a black box and a blue box, respectively. **b** Phylogenetic analysis of entire amino acid sequences deduced from MaMybA, MaAN2, and other anthocyanin-related R2R3-MYBs. The phylogenetic tree was constructed by the maximum-likelihood method using MEGA 6.0 software. The numbers next to the nodes indicate bootstrap values from 1000 replicates. Human cMYB (M15024) was used as a rooting outlier. R2R3-MYB protein sequences of different plant species were retrieved from the GenBank database, and their GenBank accession numbers are listed in brackets. **c** Expression profile of *MaMybA* in different tissues of *Muscari armeniacum*; *MaActin* was the reference gene. Error bars indicate the standard deviations (SD) of average results

Sequence alignment showed that the highly conserved MYB domain (i.e., the R2R3 repeats) is present in the N-terminal of MaMybA, MaAN2, and other anthocyanin-related R2R3-MYBs (Fig. 1a). In addition, a conserved motif [D/E]LX_2_[R/K]X_3_LX_6_LX_3_R, which is necessary to the interaction of the MYB domain with the R-like bHLH proteins [24], was present in the R3 repeat of MaMybA, MaAN2, and other anthocyanin-related R2R3-MYBs (Fig. 1a). When considering the entire amino acid sequence, MaMybA showed only 43.5% shared identity with MaAN2, 47.3% shared identity with EgVIR (an R2R3-MYB controlling seed color in *Elaeis guineensis* [25], and 43.07% shared identity with AcMYB1 (an R2R3-MYB regulating the anthocyanin accumulation of bulbs in *Allium cepa*) [26] (Fig. 1a). Moreover, MaMybA and MaAN2 shared three conserved amino acids [Arg (R), Val (V), and Ala (A)] in the R2R3 repeats, and a highly conserved motif [K/R]P[Q/R]P[Q/R] in the C-terminal with MYBs in the AN2 subgroup from eudicots and some monocots (Fig. 1a).

A phylogenetic analysis indicated that MaMybA, along with MaAN2, AtPAP1, LhMYB6, and AcMYB1, was clustered into the AN2 subgroup, and was most closely associated with AcMYB1 (Fig. 1b). However, MYBs from the monocot families Poaceae and Orchidaceae were grouped into the C1 subgroup (Fig. 1b). Additionally, the relative expression of *MaMybA* in the roots, bulbs, leaves, and five different floral developmental stages (S1~S5) showed that *MaMybA* was predominantly expressed in pigmented flowers. The mRNA level of *MaMybA* was very low in the roots, bulbs, and flowers of stage S1, which was much lower than in the leaves. The *MaMybA* transcript in flowers gradually increased, peaking at stage S3, and then slightly decreased from stages S3 to S4, before showing a fall by stage S5 (Fig. 1c).

### MaMybA was localized in the cell nucleus and had transcriptional activation ability

**Fig. 2:**
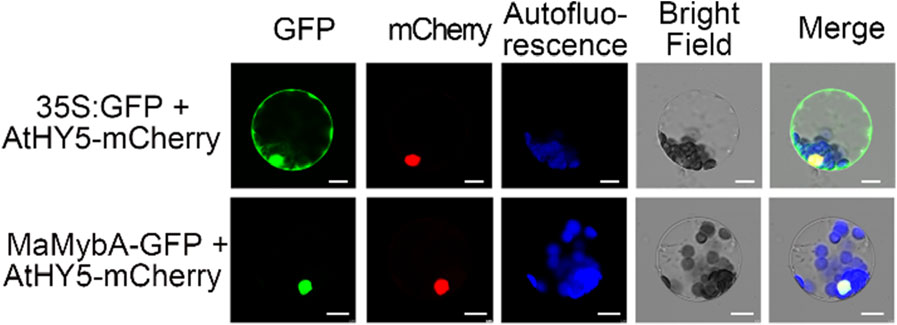
Subcellular localization of MaMybA. The transient co-expression of MaMybA-GFP with AtHY5-mCherry in *Arabidopsis thaliana* mesophyll protoplasts showed that MaMybA and AtHY5 were co-located in the nucleus. Bars: 10 μm

In order to detect the subcellular localization of MaMybA, the plasmids 35S:GFP (Green fluorescent protein) and MaMybA-GFP were separately cotransformed with AtHY5-(ELONGATED HYPOCOTYL5; AT5G11260) mCherry into *Arabidopsis* mesophyll protoplasts. The GFP fluorescence of MaMybA was visualized to overlap with the mCherry fluorescence of AtHY5, which is a known nuclear protein [27], indicating that MaMybA was localized in the nuclei of *A. thaliana* protoplasts (Fig. 2).

We performed a transactivation assay in yeast in order to assess the transcriptional activation ability of MaMybA. As shown in Additional file 1: Figure S1, the yeasts only transformed with the positive control (pGBKT7-53 + pGADT7-T), and the pGBKT7/MaMybA plasmid was able to grow in SD/-Trp media to which were added 40 μg mL^− 1^ X-α-gal and 200 ng mL^− 1^ aureobasidin A (AbA), and exhibited blue plaques. However, the negative control was not able to grow in SD/-Trp media plus 200 ng mL^− 1^ AbA or 40 μg mL^− 1^ X-α-gal and 200 ng mL^− 1^ AbA. The results showed that MaMybA has transcriptional activation ability.

### MabHLH1 interacted with MaMybA or MaAN2 in vivo

**Fig. 3:**
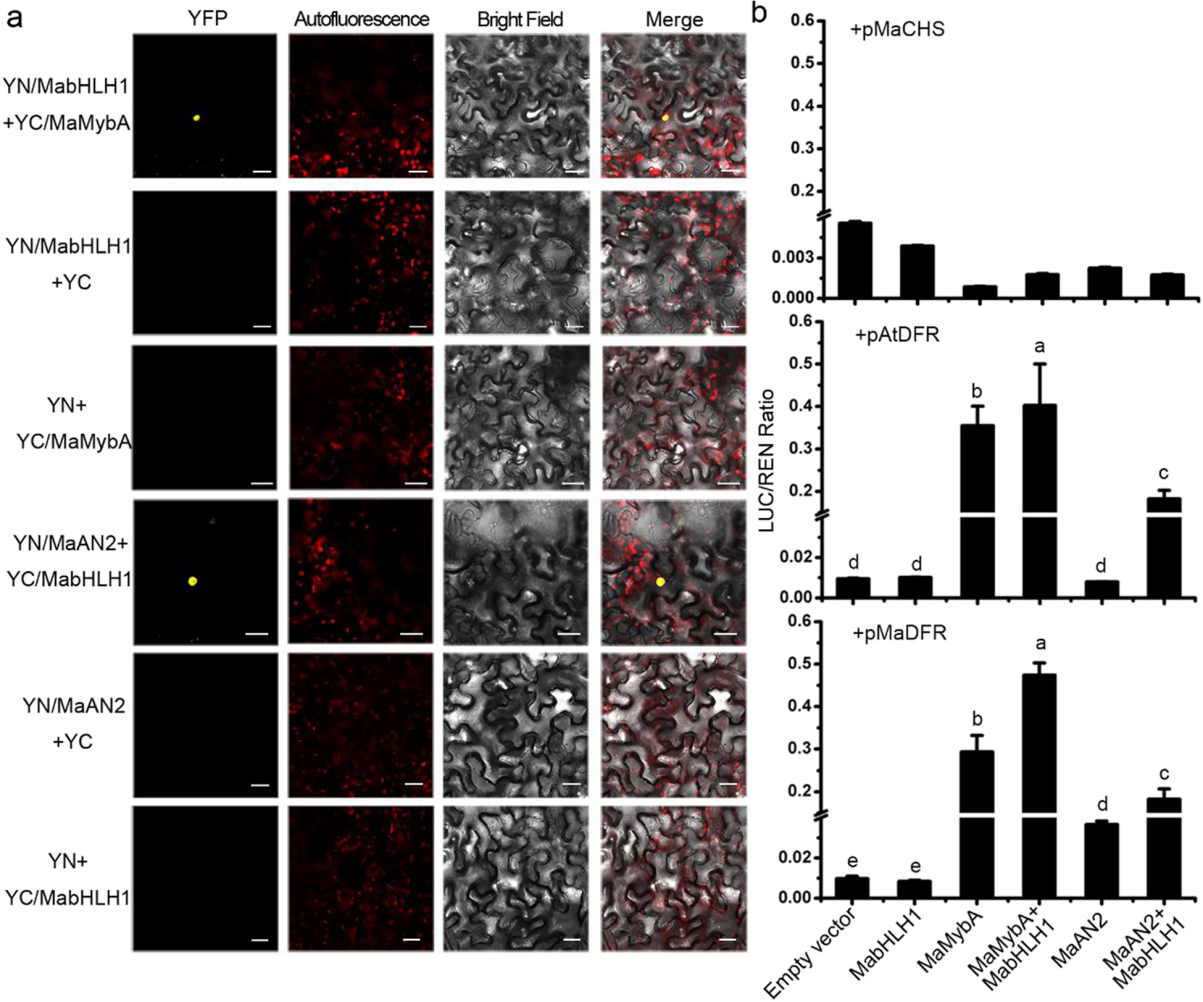
Bimolecular fluorescence complementation (BiFC) assay and dual luciferase assay. **a** BiFC assay was performed to confirm the interaction between MabHLH1 and MaMybA or MaAN2 in *Nicotiana benthamiana* leaves. Bars: 25 μm. **b** Dual luciferase assay of MabHLH1 with or without MaMybA or MaAN2: effects on the transcriptional regulation of *MaCHS*, *MaDFR*, and *AtDFR* promoters, respectively, in *N. benthamiana* leaves. The results show promoter activity expressed as the ratio of the *MaCHS*, *MaDFR*, and *AtDFR* promoters, LUC to 35S: REN. Error bars indicate the standard deviations (SD) of average results

R2R3-MYBs have been reported to interact with the IIIf bHLH protein that regulates anthocyanin biosynthesis [28–30]. We screened one anthocyanin-related bHLH unigene from the transcriptome of *M. armeniacum* with local BLASTP, using the R-like IIIf bHLH protein AtTT8. A 2001 bp full-length cDNA was obtained from *M. armeniacum* flowers using a reverse transcription-polymerase chain reaction (RT-PCR), and named *MabHLH1* (MF663729). MabHLH1 contained a highly conserved MYB interaction region in the N-terminal, and a bHLH DNA binding domain in the C-terminal (Additional file 2: Figure S2a). The phylogenetic analysis showed that MabHLH1 was grouped with LhbHLH1 and ZmLC into the LC/JAF13/DEL clade of the IIIf subgroup of the flavonoid-related bHLH proteins (Additional file 2: Figure S2b), and was also constitutively expressed in different tissues of *M. armeniacum* (Additional file 2: Fig. S2c). Subcellular localization and transcriptional activation ability assays showed that MabHLH1 was localized in the nucleus and had transactivation ability, thus meaning it was a TF (Additional file 3: Figure S3). A bimolecular fluorescence complementation (BiFC) assay was then conducted to verify the interaction between MabHLH1 and MaMybA or MaAN2 in vivo (Fig. 3a). Co-expressing either YC/MaMybA with YN/MabHLH1 or YC/MabHLH1 with YN/MaAN2 in *Nicotiana benthamiana* leaves revealed yellow fluorescent protein (YFP) fluorescence in the epidermal cell nuclei (Fig. 3a). Thus, MabHLH1 was shown to be an interacting protein from the anthocyanin-related R2R3-MYBs in *M. armeniacum*, which was able to interact with MaMybA or MaAN2 in vivo.

### MaMybA activated the anthocyanin biosynthetic gene promoters

In order to investigate whether MaMybA or MabHLH1 is able to control expression of the anthocyanin biosynthetic genes, a dual-luciferase assay was performed on *N. benthamiana* leaves (Fig. 3b). The promoters of the anthocyanin early biosynthetic gene (EBG) *MaCHS* (*M. armeniacum*; KY781171) and the key LBGs, *AtDFR* (*A. thaliana*; AT5G42800) and *MaDFR* (*M. armeniacum*; KY781169), were fused to LUC (firefly luciferase). *MaMybA*, *MaAN2*, and *MabHLH1* were driven by 35S promoters. Each or both of the TFs were co-infiltrated with each promoter. The results showed that single or both of the TFs could not increase the promoter activity of *MaCHS*. However, MaMybA alone could activate the *MaDFR* and *AtDFR* promoters, while neither MaAN2 nor MabHLH1 were able to activate both. When co-expressing MaMybA with MabHLH1, the promoter activities of *MaDFR* and *AtDFR* were significantly promoted, by 1.6 and 1.1 times higher than when infiltrated with MaMybA alone, respectively (Fig. 3b). When co-infiltrating MaAN2 with MabHLH1, the promoter activities of *MaDFR* and *AtDFR* were also significantly promoted, 4.0 and 22.2 times higher, when compared with MaAN2 infiltrated alone, respectively (Fig. 3b). Therefore, the results indicated that, MaMybA alone or in interaction with MabHLH1, could control the transcription of *MaDFR*, but MaAN2 required a direct interaction with MabHLH1 to do so.

### Heterologous expression of *MaMybA* in tobacco strongly promoted anthocyanin accumulation

**Fig. 4:**
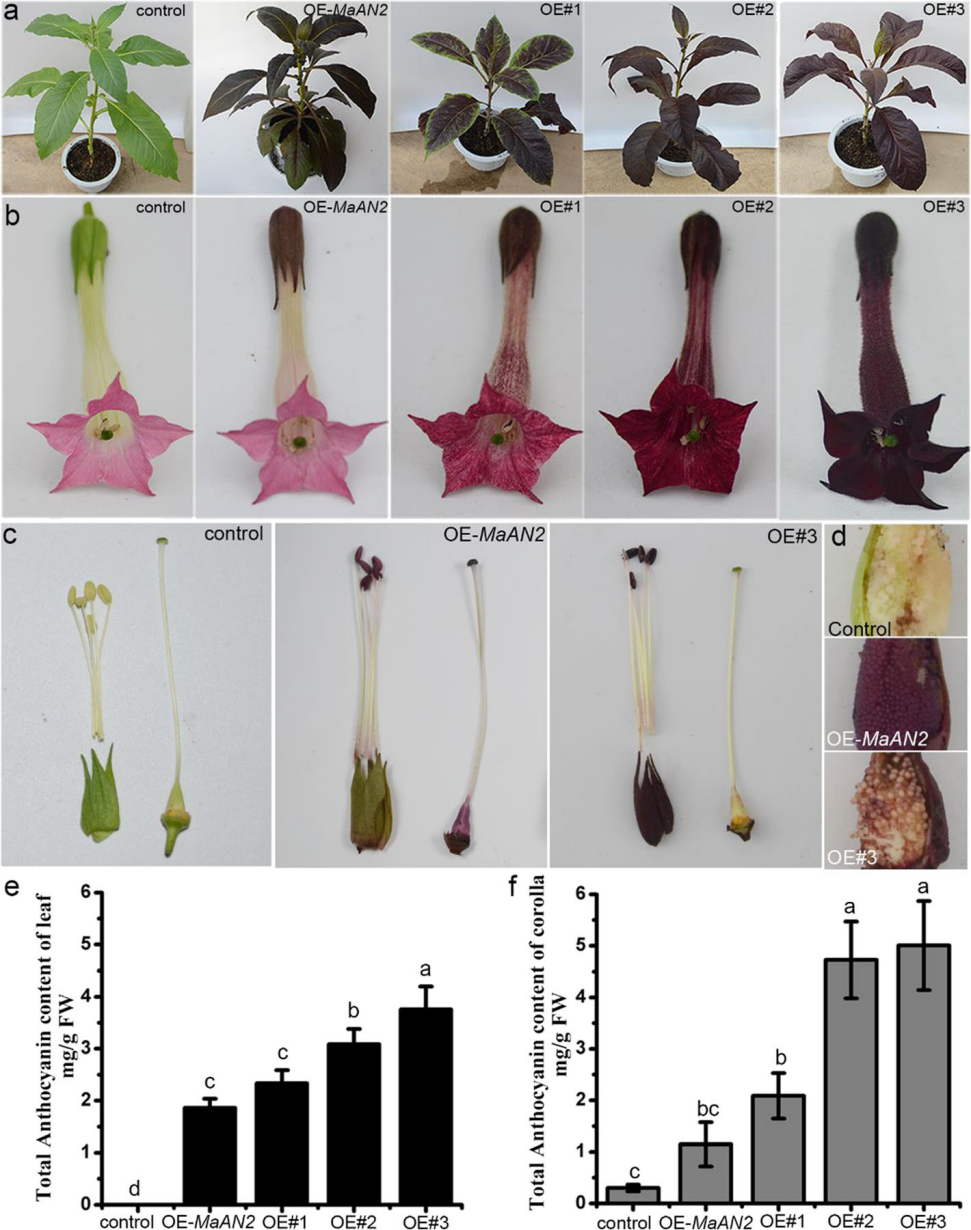
Phenotypic observation and total anthocyanin content of the leaves and corollas of the control, OE (overexpressing)-*MaAN2*, and three OE-*MaMybA* line (OE#1, OE#2, and OE#3) tobaccos. Phenotype of total plant (**a**), phenotype of flower (**b**), calyx, anthers, filament, and ovary of a flower (**c**), and seeds (**d**). A quantitative determination of the anthocyanin content of the leaves (**e**) and corollas (**f**) in the control, OE*-MaAN2*, and three OE-*MaMybA* line tobaccos. The anthocyanin extracts from the leaves and corollas were analyzed using high-performance liquid chromatography and were each monitored at 530 nm. A standard curve of cyanidin 3-rutinoside content was used as a control to calculate the anthocyanin content in the tobacco. Error bars indicate standard deviations (SD) in the average anthocyanin content

In order to characterize the function of *MaMybA*, *MaMybA* driven by a 35S promoter was transformed into tobacco (*N. tabacum* ‘NC89’) leaf discs by an *Agrobacterium*-mediated transformation. Three T_1_ overexpressing *MaMybA* (OE-*MaMybA*) transgenic lines, designated as OE#1, OE#2, and OE#3, were generated (Fig. 4a, b). Additionally, the T_1_ overexpressing *MaAN2* tobacco (the most pigmentation line (i.e., line 2) among three OE-*MaAN2* transgenic plants in respect to Chen et al. [17]) was used as the positive control (OE-*MaAN2*), and the empty vector transgenic plant was used as the negative control (control). The results showed that MaMybA strongly increased anthocyanin production and conferred an intense red-purple color in tobacco, especially in the leaf, calyx, petal, and anther (Fig. 4a-c), while the ovary wall and seed skin were barely anthocyanin pigmentation (Fig. 4c, d). However, OE-*MaAN2* tobacco presented a much lighter magenta color in the leaf, calyx, and anther than did OE-*MaMybA* tobacco; however, the ovary wall and seed skin were both pigmented with a reddish color, and the corolla was deep pink (Fig. 4a-d).

**Fig. 5:**
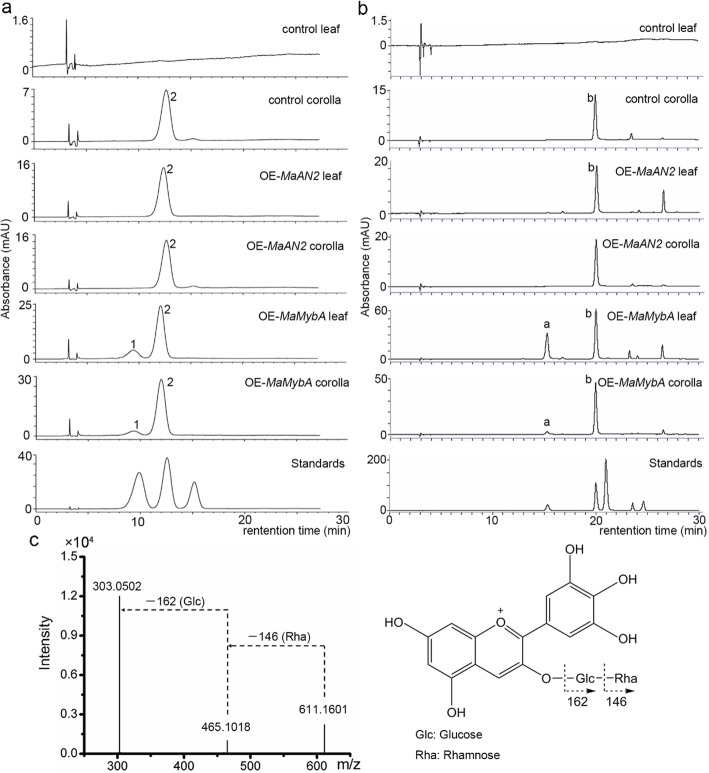
Anthocyanin composition analysis of the leaves and corollas of the control, OE-*MaAN2*, and OE-*MaMybA* tobaccos. High-performance liquid chromatography analysis of anthocyanin extract (**a**), and its corresponding hydrolysate (**b**) in the leaves and corollas of the control, OE-*MaAN2*, and OE-*MaMybA* tobaccos. According to the chromatographic peak in the sequence, the standards are delphinidin 3-rutinoside, cyanidin 3-rutinoside, and pelargonidin 3-rutinoside (from left to right) in diagram **a**; and delphinidin, cyanidin, petunidin, pelargonidin, and malvidin (from left to right) in diagram **b**. The mass spectrum and structure patterns of delphinidin 3-rutinoside (**c**)

Floral color is related to the internal or surface tissue morphology of floral organs, and to the type and content of anthocyanin pigments in the floral cells; pigment, however, is the key determinant [1]. We first determined the content and type of anthocyanin in tobaccos using high-performance liquid chromatography (HPLC). The anthocyanin concentration in the leaves and corollas of three OE-*MaMybA* tobaccos was obviously higher than in the OE-*MaAN2* and control tobaccos (Fig. 4e, f). Specifically, the anthocyanin level in three lines of OE-*MaMybA* leaves was 1.25- to 2-fold higher than that of OE-*MaAN2* leaves. The level of anthocyanin in three lines of OE-*MaMybA* corollas was 1.8- to 4.3-fold higher than that of OE-*MaAN2* corollas, and was 6.9- to 16.7-fold higher than that of the control corollas (Fig. 4e, f). Anthocyanins were virtually undetectable in the control tobacco leaves (Fig. 4e, Fig. 5a, Additional file 4: Figure S4). Next, we detected two main anthocyanins (peaks 1 and 2) in the leaves and corollas of three OE-*MaMybA* lines (OE#3 as the representative in Fig. 5a; Additional file 4: Figure S4), while primarily a single anthocyanin (peak 2) was detected in the corollas of the control and three OE-*MaAN2* lines mentioned in Chen et al. [17] (line 2 as the representative in Fig. 5a, Additional file 4: Figure S4).

Previous studies have shown the primary anthocyanin in tobacco to be cyanidin 3-rutinoside (Cy3R) [31]; the retention times of the delphinidin 3-rutinoside (Dp3R) and Cy3R standards were very close to peaks 1 and 2 (Fig. 5a), respectively; and Dp (peak a) and Cy (peak b) were detected by hydrolysis (Fig. 5b). Therefore, we made a preliminary speculation that peaks 1 and 2 represented Dp3R and Cy3R, respectively. Moreover, the two anthocyanins were confirmed by ultra-performance liquid chromatography (UPLC)-time-of-flight (TOF)-tandem mass spectrometry (MS/MS) analysis. In detail, peak 1 showed typical molecular ions at a mass-to-charge ration (m/z) of 611.1601 [M]^+^ (C_27_H_31_O_16_), which was consistent with the molecular weight of Dp3R. In addition, two product ions, one at m/z 465.1018 and one at m/z 303, indicated that one molecule of glucose and one of rhamnose were released from one molecule of Dp (Fig. 5c). Additionally, the metabolite was also used to search the SciFinder and Reaxys databases. Peaks 1 and 2 were therefore identified as representing Dp3R and Cy3R (Additional file 5: Figure S5), respectively.

Additionally, microscopic observations indicated that no anthocyanin was found in any of these cell types in the control tobacco (Additional file 6: Figure S6a-d). Anthocyanin accumulated primarily in the palisade parenchyma, abaxial epidermal cells, and trichomes in OE-*MaAN2* and OE-*MaMybA* tobacco leaves (Additional file 6: Figure S6e-l). Anthocyanin was also scattered in the adaxial epidermal cells, spongy parenchyma, and hypodermal parenchyma cells (Additional file 6: Fig. S6 g, h, k, l). Moreover, there were no morphological changes in the epidermal cells of leaves and corollas between the control, OE-*MaAN2*, and OE-*MaMybA* plants, according to scanning electron microscopy (Additional file 7: Figure S7).

### Overexpression of *MaMybA* in tobacco upregulated the expression levels of anthocyanin pathway genes

**Fig. 6:**
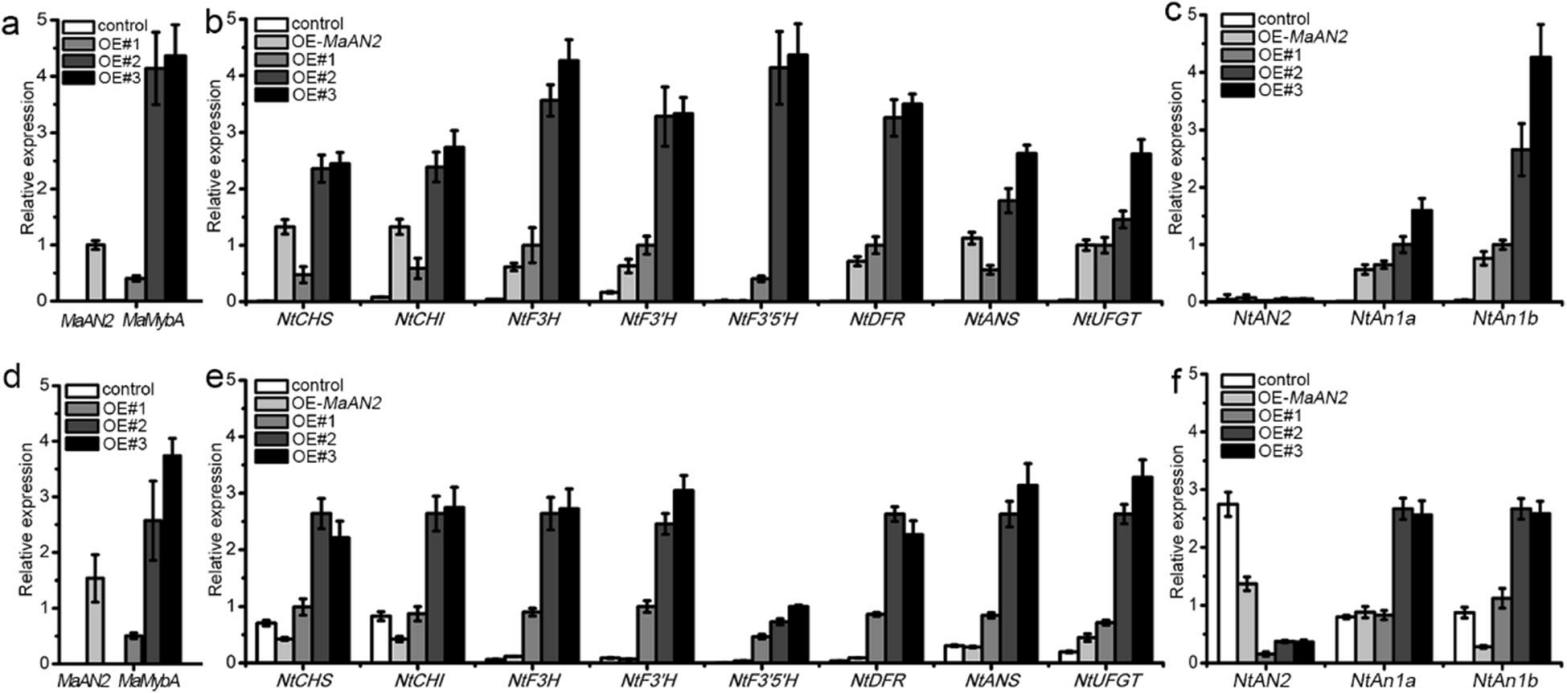
Relative expression of anthocyanin biosynthetic genes and endogenous transcription factor (TF) genes in the leaves and corollas of the control, OE-*MaAN2*, and three independent OE-*MaMybA* transgenic plants (OE#1, OE#2, and OE#3). The expression profiles of *MaMybA* in the leaves (**a**) and corollas (**d**) of the control, OE#1, OE#2, and OE#3; the expression profile of *MaAN2* in the leaves (**a**) and corollas (**d**) of the OE-*MaAN2* tobacco; the expression levels of anthocyanin biosynthetic genes (*NtCHS*, *NtCHI*, *NtF3H*, *NtF3′H*, *NtF3′5′H*, *NtDFR*, *NtANS*, and *NtUFGT*) in the leaves (**b**) and corollas (**e**) of different tobacco genotypes; and the relative expression of three endogenous TF genes (*NtAN2*, *NtAn1a* and *NtAn1b*) in the leaves (**c**) and corollas (**f**) of different tobacco genotypes. *NtTubA1* was used as the internal control. Error bars indicate the standard deviations (SD) of average results

We first performed a quantitative real-time polymerase chain reaction (qRT-PCR) assay to test *MaMybA* transcripts in the transgenic tobaccos, and found that *MaMybA* was expressed in the leaves and corollas of three transgenic lines (OE#1, OE#2, and OE#3), but not in the negative control plants (i.e., control, Fig. 6a, d). In addition, *MaAN2* was also expressed in the positive control tobacco (i.e., OE-*MaAN2*, Fig. 6a, d). Next, we conducted a qRT-PCR assay of anthocyanin biosynthetic and TF genes in the leaves and corollas of the control, OE-*MaAN2*, and OE-*MaMybA* tobacco. The results showed that all the anthocyanin structural genes, including *NtCHS*, *NtCHI*, *NtF3H*, *NtF3′H, NtF3′5′H, NtDFR*, *NtANS*, and *NtUFGT*, were upregulated in the leaves and corollas of transgenic plants, especially in OE#2 and OE#3 (Fig. 6b, e). The two endogenous bHLH TF genes *NtAn1a* and *NtAn1b*, which regulate flavonoid synthesis in tobacco [32], were also upregulated in the leaves and corollas of the OE-*MaMybA* plants (Fig. 6c, f). However, the transcripts of *NtAN2* were barely detected in the leaves of all genotypes of tobaccos, and those in the corollas of OE-*MaAN2* and OE-*MaMybA* plants were lower than those in the control (Fig. 6c, f).

## Discussion

Anthocyanins are the primary pigments in flowers. Anthocyanin biosynthesis is regulated by R2R3-MYB TFs, or the MBW complex [33]. R2R3-MYB TFs involved in flower coloration have been found in various ornamental plants, including *Gerbera hybrida*, *Lilium*, and *Anthurium* [14–16]. In grape hyacinth, previous study has shown that an anthocyanin-related R2R3-MYB, MaAN2, induced the obvious increment of cyanidin biosynthesis in tobacco and relied on AtTT8 or MabHLH1 to activate the LBG expression [17] (Fig. 3b). In the present study, we isolated a novel R2R3-MYB, MaMybA, which differed from MaAN2. Heterologous expression of *MaMybA* conferred intense and dark anthocyanin pigmentation in tobacco, and produced a new anthocyanin, Dp3R. The study determined some precise differences between MaMybA and MaAN2 functioning in anthocyanin biosynthesis, and further revealed the regulatory mechanism responsible for coloration in *M. armeniacum* flowers.

### MaMybA is an R2R3-MYB TF in the AN2 subgroup of the R2R3-MYB family

Anthocyanin-promoting R2R3-MYB TFs have specific structures and typical motifs. In *M. armeniacum*, we characterized an R2R3-MYB MaMybA, similar to MaAN2, which contained three conserved amino acids (Arg, Val, and Ala) in the highly conserved R2R3 domain of N-terminal region and a typical motif [K/R]P[Q/R]P[Q/R] in the C-terminal region. These conserved amino acids and typical motif have also been reported as present in anthocyanin-promoting R2R3-MYBs of the AN2 subgroup in dicots [7, 11, 20] and a few monocots [15–17, 34] (Fig. 1a, b). Notably, MaMybA and MaAN2 in monocot plants were not found to contain the motif KAX[K/R]C[S/T] in the C1 subgroup of R2R3-MYBs from the Poaceae and Orchidaceae plant families (Fig. 1a, b). Additionally, the motif [D/E]LX_2_[R/K]X_3_LX_6_LX_3_R in the R3 repeat of MaMybA and MaAN2 (Fig. 1a) indicated that MaMybA and MaAN2 could interact with bHLH TFs. MaMybA was identified as a TF because it was localized in the cell nucleus, and had transcriptional activation ability (Fig. 2, Additional file 1: Figure S1).

To our knowledge, all anthocyanin-related dicot R2R3-MYB TFs have been grouped into the AN2 subgroup of the R2R3-MYB family. In monocots, only those MYBs in Poaceae and Orchidaceae are clustered into the C1 subgroup [30, 35, 36]; the other monocot MYBs are placed in the AN2 subgroup [16, 17, 25, 26, 34]. Therefore, more anthocyanin-related R2R3-MYBs should be excavated in order to determine the regularity of clustering between dicots and monocots.

Interestingly, phylogenetic analysis indicated that the evolutionary relationship of MaMybA was closer than that of MaAN2 to AcMYB1. MaMybA and MaAN2 shared an identity of only 43.5% in their whole amino acid sequences. The relative expression of *MaMybA* in different tissues indicated that it was predominantly expressed in pigmented flowers (Fig. 1c). Its expression was coordinated with, although slightly earlier than that of *MaAN2*, and also with pigment accumulation during flower development [17].

The large family of R2R3-MYB is the major family of MYB proteins. R2R3-MYBs all have the highly conserved R2R3 domain at the N-termini, which decide the regulatory specificity and offer the MYB nuclear localization signals, while the C-terminal regions often possess transactivation or DNA-repression motifs [37, 38]. In *Arabidopsis*, the R2R3-MYBs are divided into 22 subgroups according to the recurring amino acid motifs in the C-termini [39]. Specifically, the R2R3-MYBs of AN2 subgroup (i.e., subgroup 6) which consist of the conserved motif ([K/R]P[Q/R]P[Q/R]) in the C-terminal regions function in inducing anthocyanin biosynthesis [34]; the R2R3-MYBs of subgroup 4 contained the conserved EAR motif (LxLxL) in the C-termini function in reducing anthocyanin biosynthesis [40]. Previous study also showed that R2R3-MYBs which contain highly conserved motifs and exon lengths have similar functions between *Arabidopsis* and *Vitis*, although low sequence homology in the C-terminal regions of all R2R3-MYB proteins. Thus, despite occurrence of diversification, the highly conserved regions could maintain the specific functionality, while variations in C-terminal regions could facilitate the gaining of new or cooperative functions [37].

In planta, there are numerous examples where two or more R2R3-MYB genes, functioning in anthocyanin biosynthesis, are found within a single plant species, such as AtPAP1 and AtPAP2 in *Arabidopsis* [20]. However, some R2R3-MYB anthocyanin regulators found in the same plant show diversity through functioning in anthocyanin pigmentation in different tissues or organs; for example, *Petunia* AN2 is responsible for flower limb pigmentation, while AN4 mainly regulates anther anthocyanin accumulation [41]; in *Solanum lycopersicum*, two different R2R3 MYB, SlANT1 and SlAN2, both could induce anthocyanin biosynthesis, but only the latter one functions in anthocyanin synthesis in vegetative tissues under high light or low temperature conditions [42]. In grapevine, four anthocyanin-related R2R3 MYBs have different amino acid lengths of C-termini. Particularly, VvMYBA2 has two C-terminal repeats, while VvMYBA1 has one and VvMYBA3 lacks this motif. Besides, MYBA1 and MYBA2 function fruit coloration, but MYBA3 loses the function of accumulating anthocyanin. These differences could cause novel or cooperative functions [43]. In this study, each of MaMybA and MaAN2 only contained one conserved motif (KPQPQ) in the C-terminal region. Furthermore, premature termination of the C-terminal domain did not occur in MaMybA or MaAN2 (the amino acid lengths of MaMybA and MaAN2 were 237 and 240, respectively), and the two MYBs could perform complete functions in anthocyanin biosynthesis in tobacco. Thus, MaAN2 might be the paralog of MaMybA by gene duplication in the process of evolution, and the variation in C-terminal indicated that they might possess redundant, overlapping or cooperative functions in grape hyacinth. So, based on this, the experimental evidence on functionality of the variable C-terminal between MaMybA and MaAN2 is a future direction of our work.

### MaMybA alone or in interaction with MabHLH1 could activate anthocyanin biosynthetic gene promoters

Anthocyanin biosynthesis is regulated by MYB-bHLH complexes in almost all plant species [33]. In the present study, the resulting MabHLH1 contained an MYB interaction region on the N-terminal and a bHLH DNA binding domain at the C-terminal region, together with other IIIf bHLH proteins involved in flavonoid biosynthesis [28, 30, 44] (Additional file 2: Figure S2a). The phylogenetic analysis indicated that MabHLH1 was grouped into the LC/JAF13/DEL clade of the IIIf bHLH TF family [26, 45] (Additional file 2: Figure S2b). These findings indicated that MabHLH1 might play a role in anthocyanin biosynthesis in *M. armeniacum*. However, *MabHLH1* was constitutively expressed in different tissues, and was not concomitant with the pigmentation of flower development (Additional file 2: Figure S2c), which has also been found in apples [46], grapes [47], and *Phalaenopsis* [36].

MabHLH1 interacted with either MaMybA or MaAN2 in vivo; this was confirmed by the BiFC assay (Fig. 3a). In the dual luciferase assay, we found that the promoter activity of *MaCHS* (EBG) was induced by none of all TFs (each or both of MYB-bHLH complexes) in this assay, which was consistent with the result that the activity of *MrCHS* promoter was not response to MrMYB1, or MrbHLH1, or MrMYB1-MrbHLH1 in *Myrica rubra* [48]. MaMybA alone was able to regulate the expression of *MaDFR* (LBG), in contrast to MaAN2, which relied on MabHLH1 to control *MaDFR* expression (Fig. 3b). In maize, ZmP alone can activate flavonoid biosynthetic genes, but ZmC1 require a direct interaction with bHLH protein R/B to do so [49]; in *Phalaenopsis*, PeMYB11 is dependent on PebHLH1, while PeMYB2 and PeMYB12 are independent of PebHLH1 in regulating *PeDFR* expression [36]. The regulation pattern of ZmP, PeMYB2, and PeMYB12 is consistent with that of MaMybA. However, in *Arabidopsis*, AtPAP1 interacts with AtTT8 or AtGL3 to regulate *AtDFR* expression [50, 51]. PhAN2 also requires bHLH cofactor PhAN1 or PhJAF13 to enhance promoter activity in *Petunia PhDFR* [44]; and the MdMYB10-MdbHLH3 and MdMYB10-MdbHLH33 complexes can each activate the expression of *MdDFR* in apples [28]. These regulation patterns are highly consistent with that of MaAN2.

### Phenotypic differences between OE-*MaMybA* and OE-*MaAN2* tobacco can be ascribed to the high concentration of anthocyanin and the newly produced Dp3R in OE-*MaMybA* tobacco

The ectopic expression of a gene in a model plant quickly facilitates its functional identification, and visible anthocyanin pigmentation could directly demonstrate its function in anthocyanin biosynthesis [52]. Many studies show that overexpressing anthocyanin-related R2R3-MYB genes could promote anthocyanin production in tobacco by genes such as *AtPAP1* [18, 20], and *AaMYB2* [16]. In this study, overexpressing *MaMybA* in tobacco induced a high level of anthocyanin production in the leaf, petal, anther, and calyx, which all presented with mulberry pigmentation (Fig. 4a-c), while the ovary wall and seed skin were barely pigmented (Fig. 4c, d). However, OE-*MaAN2* tobacco presented a magenta leaf, calyx, anther, and a deep pink corolla, which was much lighter than in OE-*MaMybA* tobacco (Fig. 4a-c); the ovary wall and seed skin of OE-*MaAN2* tobacco showed a reddish color, which were different from that of OE-*MaMybA* (Fig. 4c, d). A microscopic observation of OE-*MaMybA* tobacco leaves indicated that anthocyanin was primarily present in the palisade parenchyma, trichomes, and lower epidermis, with some pigment scattered in the upper epidermis and spongy mesophyll; and the pigmentation was much deeper in comparison with OE-*MaAN2* transgenic plants (Additional file 6: Figure S6). In OE-*AtPAP1* tobacco, the pigmentation cells were primarily found in the trichomes and lower epidermis [18]. Thus, different R2R3-MYBs influence anthocyanin accumulation in different tissues or cells to various extents, and the distribution of anthocyanin might affect plant color.

Previous studies have demonstrated that the primary anthocyanin in the tobacco corolla is Cy3R [31]. In the present study, a newly produced anthocyanin, Dp3R, was found in the leaves and corollas of OE-*MaMybA* tobacco, in addition to Cy3R, the primary anthocyanin (Fig. 5a); this differed from OE-*MaAN2* tobacco, in which the primary anthocyanin in the leaves and corollas was found to be Cy3R (Fig. 5a). Additionally, the anthocyanin levels of the leaves and corollas in three OE-*MaMybA* transgenic lines were all greater than 2.0 mg g^− 1^ fresh weight (FW), while those in OE-*MaAN2* plants were both lower 2.0 mg/L FW (Fig. 4e, f). Previous study has shown that two anthocyanin regulatory genes (*Arabidopsis* R2R3 MYB gene *mPAP1* and *Zea mays* bHLH-type gene *B-Peru*) overexpressing in *N. tabacum* L. to yield an enhanced anthocyanin content, and also produced a new type pigment Dp3R in the flower limb of transgenic tobacco, which presented a dark red color [53]. The hydrolysate of anthocyanin extracts from the leaves and corollas of OE-*MaMybA* tobacco also showed that Cy was the primary anthocyanidin, followed by Dp (Fig. 5b); this was similar to the results of anthocyanin hydrolysis from OE-*AtPAP1* tobacco [18]*.* Previous studies have shown that the reddish-purple flower of grape hyacinth (*M. comosum* ‘Plumosum’) contains two main anthocyanidins (Dp and Cy) that contribute to its color [22]. Therefore, we concluded that the red Cy-based derivatives, together with the blue Dp-based derivatives and the high anthocyanin concentration, contributed to the dark reddish-purple color in OE-*MaMybA* tobacco. Moreover, when overexpressing different R2R3-MYB genes in model plants, the resultant plant phenotypes were distinct in anthocyanin localization, type and content, possibly because different R2R3-MYB TFs regulate different target genes to induce different anthocyanins in different tissues.

R2R3-MYB proteins of MBW complexes play core roles in anthocyanin accumulation, and the MBW complexes could probably induce the entire anthocyanin biosynthesis pathway [8]. In the present study, expression levels of almost all the anthocyanin biosynthetic genes (*NtCHS*, *NtCHI*, *NtF3H*, *NtF3’H*, *NNtDFR*, *NtANS*, and *NtUFGT*) and endogenous bHLH TF genes (*NtAn1a* and *NtAn1b*) were upregulated in the leaves and corollas of three OE-*MaMybA* transgenic lines (especially in OE#2 and OE#3; Fig. 6); this was consistent with the results for the leaves of OE-*AaMYB2* and OE-*MaAN2* tobacco [16, 17] (Fig. 6b, c). However, the transcripts of *NtAN2* were not detected in the leaves of all detected tobaccos (Fig. 6c), because NtAN2 is a floral tissue-specific anthocyanin-related R2R3-MYB in *N. tabacum* [54]. Moreover, the expression levels of *NtAN2* in the corollas of transgenic plants were lower than that in control (Fig. 6f); this was similar to the results of the overexpressing *B-peru* and *mPAP1* in tobacco, which indicated that excess anthocyanin pigmentation could inhibit the expression of endogenous gene by feedback regulation [53]. Notably, *NtF3′5′H* transcripts in the leaves and corollas of OE-*MaMybA* tobacco were much higher than in the control and OE-*MaAN2* plants (Fig. 6b, e). These results indicated that MaMybA promoted anthocyanin accumulation by upregulating the anthocyanin biosynthetic genes in the synthesis pathway. These upregulated anthocyanin biosynthetic genes led to an increase in metabolites, which formed the substrates for the subsequent catalytic reaction and therefore enabled the accumulation of a high concentration of anthocyanins. Additionally, the highly expressed *NtF3′5′H* was responsible for catalyzing the product of Dp3R that was also found in the flower limbs of transgenic tobacco in previous study [53].

## Conclusions

In this study, a novel R2R3-MYB TF, MaMybA, which was identified from the flower transcriptome of *M. armeniacum*, was found to play a role in anthocyanin pigmentation. We determined the function of MaMybA in anthocyanin biosynthesis by heterologous expression in tobacco, and that the intense and dark reddish-purple anthocyanin pigmentation of OE-*MaMybA* tobacco could be attributed to the high concentration of anthocyanin and the newly produced Dp3R. The results indicated that *MaMybA* might be a candidate gene for modifying flower color in ornamental plants and potentially in engineering high levels of anthocyanins. Moreover, we also determined the similarities and differences in anthocyanin synthesis between MaMybA and MaAN2: they both could interact with a bHLH protein, but the former was independent whilst the latter was dependent of the bHLH to regulate LBGs. Besides, MaMybA was different from MaAN2 in inducing the content and composition of anthocyanin and coloring tissues in tobacco, which suggested that future work focuses on their functions in grape hyacinth.

## Methods

### Plant materials

Grape hyacinth (*M. armeniacum*) plants were grown in an experimental field at Northwest A&F University in Yangling District, Shaanxi province, China. The tepals were divided into five stages (S1~S5) mainly according to the degree of petal pigmentation: S1 indicated no pigmentation; S2 showed pigmentation visible on the basal part; S3 indicated pigment beginning to turn blue; S4 presented that the flowers were completely blue, but had not opened; and S5 indicated that flowers completely opened, according to the method of Chen et al. [17], and were then collected and stored, together with the vegetative tissues (roots, bulbs, and leaves), at − 80 °C until use. Aseptic tobacco (*N. tabacum* ‘NC89’) seedlings were used for genetic transformation at the four-leaf stage. The T_1_ generation transgenic tobacco plants were transferred from an aseptic culture room to a greenhouse. The mature, fully expanded tobacco leaves and fully opened flower limbs were picked and stored at − 80 °C in readiness for the next test. *Arabidopsis thaliana* (Col-0) and *N. benthamiana* plants were grown in a light incubator under a 16 h light/8 h dark cycle until the four to six-leaf stage was achieved for cloning the *AtDFR* promoter and *AtHY5* gene, and for dual luciferase assay, respectively.

### Quantitative real-time PCR assay

Total RNA extraction from different tissues of *M. armeniacum* and tobacco, cDNA synthesis, and a qRT-PCR assay were undertaken using the protocols described by Chen et al. [17]. The qRT-PCR primers are listed in Additional file 8: Table S1. *MaActin* and *NtTubA1* were used as the internal control genes for *M. armeniacum* and tobacco, respectively. Analysis was performed using three samples, with three replicates.

### Gene cloning

Local BLASTP software was used to screen one anthocyanin-related R2R3-MYB and one anthocyanin-related bHLH unigene, using AtPAP1 and AtTT8 from a transcriptome of *M. armeniacum* flowers [23]; the unigenes were designated *MaMybA* and *MabHLH1*, respectively. However, the R2R3-MYB unigene lacked the 5′- and 3′-untranslated regions. Thus, we used the 5′-RACE and 3′-RACE methods to obtain the full-length cDNA sequence of *MaMybA* from *M. armeniacum* flowers. A detailed protocol for obtaining the full-length cDNA of *MaMybA* was followed in accordance with the methods described by Chen et al. [17] and Huang et al. [55]. The full-length cDNA of *MabHLH1* was cloned from *M. armeniacum* flowers using RT-PCR; the primers used for gene cloning are listed in Additional file 8: Table S1. The resulting cDNA sequences were submitted to the NCBI GenBank database under the accession numbers MF663728 (*MaMybA*) and MF663729 (*MabHLH1*).

### Sequence alignment and phylogenetic analysis

The deduced amino acid sequences of *MaMybA* and *MabHLH1*, as well as the other anthocyanin-related R2R3-MYBs and bHLH proteins retrieved from the GenBank database, were used for sequence alignment and phylogenetic analysis, respectively. The multiple sequence alignment was analyzed using DNAMAN software version 8.0 (Lynnon Biosoft, CA, USA). The phylogenetic tree was first analyzed using CLUSTALW software and then constructed with MEGA software version 6.0 [56], using the maximum-likelihood method. The bootstrap was set at 1000 replicates. The accession numbers of the different protein types are listed in Fig. 1 and Additional file 2: Figure S2, respectively.

### Subcellular localization and transcription activation ability assay

For the subcellular localization assay, the ORF of either *MaMybA* or *MabHLH1*, without a termination codon, was inserted into pCambia2300 (pC2300)-GFP using a Seamless Cloning and Assembly Kit (Novoprotein, Shanghai, China) to generate either pC2300-MaMybA-GFP (MaMybA-GFP) or pC2300-MabHLH1-GFP (MabHLH1-GFP), respectively. Additionally, the full-length cDNA sequence of *AtHY5* (AT5G11260), which encodes a nuclear localization marker protein, was cloned from the leaves of *A. thaliana* (Col-0) and inserted into pBI221-mCherry to generate pBI221-AtHY5-mCherry (AtHY5-mCherry). The primers are listed in Additional file 8: Table S1. pC2300-GFP (35S:GFP) was used as a positive control. The various plasmids were cotransformed into *A. thaliana* mesophyll protoplasts using the protocol described by Yoo et al. [57]. The transformed protoplasts were cultivated for 16 h and then visualized using a laser scanning confocal microscope (Leica TCS SP8, Wetzlar, Germany).

In order to construct yeast expression vectors for measuring the transcription activation ability of MaMybA and MabHLH1, the two ORF PCR products were separately fused into pGBKT7. The primers are listed in Additional file 8: Table S1. pGBKT7-53 was co-transformed with pGADT7-T as a positive control, and pGADT7 was used as a negative control. The yeast transformation and autoactivation testing were each performed as described in Chen et al. [17].

### Bimolecular fluorescence complementation assay

In order to verify the interaction between bHLH protein and R2R3-MYBs from *M. armeniacum*, a BiFC assay was conducted on the leaves of *N. benthamiana* to confirm the interaction between MabHLH1 and either MaMybA or MaAN2 (anthocyanin-related R2R3-MYB in *M. armeniacum*; KY781168) [17]. The ORF sequences of *MabHLH1* and *MaAN2* were individually inserted into pSPYNE (R) 173 (YN) to generate YN/MabHLH1 and YN/MaAN2, respectively. Additionally, the ORF sequence without either the *MaMybA* or *MabHLH1* termination codon was inserted into pSPYCE (M) (YC) to generate YC/MaMybA and YC/MabHLH1, respectively. The primers are listed in Additional file 8: Table S1. The recombinant and empty vector plasmids were transformed into GV3101 by electroporation, and the various transformed *Agrobacterium* cultures were co-infiltrated into *N. benthamiana* leaves. After being cultivated in a light incubator for 3 d, the infiltrated tobacco leaves were observed and photographed using a laser scanning confocal microscope (Leica TCS SP8, Wetzlar, Germany).

### Dual luciferase assay

For the dual luciferase assay, the full-length cDNA sequences of *MaMybA*, *MaAN2*, and *MabHLH1* were each cloned into pGreenII 62-SK. The promoters of the EBG in *M. armeniacum* (*MaCHS*, KY781171) and the key LBGs in *M. armeniacum* (*MaDFR,* KY781169) and *A. thaliana* (*AtDFR*, AT5G42800) were each inserted into pGreenII 0800-LUC. The primers are listed in Additional file 8: Table S1. All the recombinant plasmids were electroporated into GV3101. Infiltration, transient expression analysis, and measurements of enzyme activity for LUC and *Renilla* luciferase (REN) were performed according to previously cited protocols [17, 58, 59]. The statistical analysis of the dual luciferase assay was conducted using four biological replicates.

### Stable tobacco transformation

In order to verify the function of *MaMybA*, the ORF sequence without termination codons for *MaMybA* was inserted into pCambia1304. The primers were listed in Additional file 8: Table S1. Thus, *MaMybA* was promoted by the 35S promoter. The recombinant or empty vector plasmid was introduced into GV3101 by electroporation. The tobacco leaf disk transformation was performed according to the protocol described by Horsch et al. [60]. The transformed tobacco plants were screened using 25 mg L^− 1^ hygromycin antibiotic, which was used as the plant selective marker. The next stage of analysis used T_1_ generation transgenic plants of overexpressing *MaMybA* (OE*-MaMybA*) that showed obvious color changes in the leaves and flowers. The method of obtaining the T_1_ generation transgenic tobaccos of overexpressing *MaAN2* was followed according to the protocols of our previous study [17]*.*

### HPLC analysis

The method for extracting anthocyanin from the fresh, mature, fully expanded tobacco leaves and fully opened flower limbs of different transgenic tobacco genotypes was conducted as previously described by Chen et al. [17]. In addition, in order to obtain anthocyanin hydrolysate, a 300 μL aliquot with an equal volume of 6 M HCl was boiled for 1 h to release anthocyanidin aglycones. All the extracts were filtered through a 0.22 μm filter membrane and then subjected to reverse HPLC analysis. The HPLC experimental conditions, instruments, and protocols were performed according to Chen et al. [17]. The anthocyanin extracts were first identified with reference to commercial standards (all the standards mentioned are listed in Fig. 5; Sigma, USA). The anthocyanin content was determined according to the standard curve of Cy3R. The anthocyanin content in each tobacco sample was analyzed using three biological replicates.

### UPLC-triple-TOF-MS/MS conditions

UPLC experiments were performed on an Acquity™ UPLC system (Waters, MA, USA) coupled with a C18 column (1.8 μm, 100 mm × 4.6 mm; Agilent ZORBAX-SB, CA, USA). The column temperature was maintained at 30 °C; the detection wavelength was 530 nm. The eluent consisted of aqueous solution A (0.1% formic acid in water) and organic solvent B (0.1% formic acid in acetonitrile) at a flow rate of 0.8 mL min^− 1^. The gradient elution programme was conducted as follows: 0~2 min, 5% B; 25 min, 50% B; 35 min, 95% B; 37 min, 95% B; and 38 min, 5% B. The injection volume was 5 μL.

Mass spectrometry (MS) was performed on a TOF mass spectrometer (Triple TOF™ 5600+ system) with a Duo-Spray™ source operating in the positive and negative electrospray ionization modes (AB SCIEX, CA, USA). The optimized operating parameters of the MS/MS detector were as follows: ion spray voltage, − 4.5 kV (negative) and 5.5 kV (positive); ion source heater, 550 °C (negative) and 600 °C (positive); curtain gas, ion source gas 1, and ion source gas 2 set to 35, 50, and 50 psi, respectively; and the experiments were run using a 100~1000 m/z scan. For the MS experiments, the declustering potential and collision energy levels were set at 100 V and 10 V, respectively; the MS/MS data were collected using the TOF MS-Product Ion-IDA (information-dependent acquisition) mode; and the collision-induced dissociation energy levels were set to − 20, − 40, and − 60 V. In addition, an automated calibration delivery system was used to simultaneously calibrate the MS and MS/MS experiments, in order to reduce the mass axis error to less than 2 × 10^− 6^.

### Histological localization of anthocyanins and scanning electron microscopy

The epidermis was peeled from the leaf using fine-tipped forceps. Cross-sections of fresh leaf tissues were made by hand using a razor blade. The samples were immediately transferred to glass slides with a drop of water and observed under a microscope (Nikon Eclipse 50i, Tokyo, Japan).

The scanning electron microscopy was performed according to the previously published protocol described by Qi et al. [61]. Specifically, the pigmented leaves and corollas of transgenic tobacco were first fixed in 4% glutaraldehyde, and then dehydrated in an alcohol series. Next, the samples were critical point-dried and sputter-coated with platinum. Lastly, all the samples were observed under a scanning electron microscope (JEOL JSM-6360LV, Tokyo, Japan).

### Statistical analysis

The statistical analysis was conducted using SPSS 20.0 software (SPSS Inc., Chicago, USA). Data are presented as means **±** standard deviations (SD). The levels of statistical significance were determined by *Least Significant Difference* (*LSD*) analysis: *P* < 0.05.

## Additional files

Additional file 1: **Figure S1.** Transcription activation ability of MaMybA. Yeasts transformed with the pGBKT7/MaMybA vector (positive control (pGBKT7-53 + pGADT7-T) and negative control (pGBKT7)), were each cultured in SD/-Trp media, SD/-Trp media with 40 mg mL^− 1^ X-α-gal, and SD/-Trp media added to 40 mg mL^− 1^ X-α-gal and 200 ng mL^− 1^ AbA. (DOCX 273 kb)

Additional file 2: **Figure S2.** Alignment and phylogenetic analysis of the deduced amino acid sequence of *MabHLH1* and other basic Helix-Loop-Helix (bHLH) proteins associated with flavonoid biosynthesis from other plants. **a** Sequence alignment of partial amino acids deduced from MabHLH1 and other bHLH proteins from other plant species. The MYB-interaction and bHLH DNA binding regions are indicated above the alignment by bold black lines. **b** Phylogenetic analysis of entire amino acid sequences deduced from MabHLH1 and other bHLH proteins from other plants. The maximum-likelihood phylogenetic tree was generated using MEGA 6.0 software. Numbers next to the nodes indicate the bootstrap values from 1000 replications. The bHLH protein sequences of different plant species were retrieved from GenBank database and their GenBank accession numbers are as followings: *Arabidopsis thaliana*: AtTT8 (NP_1927202), AtEGL3 (NP_0011853021), AtGL3 (NP_0013327061), and AtMYC1 (AEE818881); *Petunia hybrida*: PhJAF13 (AAC394551) and PhAN1 (AAG259281); *Nicotiana tabacum*: NtAN1a (AEE992571) and NtAN1b (AEE992581); *Antirrhinum majus*: AmDELILA (AAA326631); *Gerbera hybrida*: GMYC1 (CAA076151); *Vitis vinifera*: VvMYC1 (ACC686851) and VvMYCA1 (ABM923323); *Malus domestica*: MdbHLH3 (ADL365971) and MdbHLH33 (ABB844741); *Myrica rubra*: MrbHLH1 (AGO583721); *Perilla frutescens*: PfMYC-RP (BAA75513); *Brassica rapa*: BrTT8 (AEA032811); *Medicago truncatula*: MtTT8 (AKN796061); *Lotus japonicus*: LjTT8 (BAH288811); *Dahlia pinnata*: DvIVS (BAJ335151); *Ipomoea nil*: InbHLH1 (BAE943931) and InbHLH2 (BAE943941); *Fragaria×ananassa*: FabHLH3 (AFL024631) and FabHLH33 (AFL024651); *Prunus persica*: PpbHLH3 (AIE575081); *Prunus avium*: PabHLH3 (AJB284811) and PabHLH33 (AJB284841); *Litchi chinensis*: LcbHLH3 (APP941241), LcbHLH1 (APP941221), and LcbHLH2 (APP941231); *Lilium* hybrid: LhbHLH1 (BAE200571) and LhbHLH2 (BAE200581); *Zea mays*: ZmLC (NP_0011053391); *Dendrobium* hybrid: DhbHLH1 (AQS798531); *Gentiana triflora*: GtbHLH1 (BAH033871). **c** Expression profile of *MabHLH1* in different tissues of *Muscari armeniacum*. *MaActin* was the reference gene. Error bars indicate the standard deviations (SD) of average results. (DOCX 1686 kb)

Additional file 3: **Figure S3.** Subcellular localization and transcription activation ability of MabHLH1. **a** Subcellular localization of MabHLH1. The transient co-expression of MabHLH1-GFP with AtHY5-mCherry in *Arabidopsis thaliana* mesophyll protoplasts showed that MabHLH1 and AtHY5 were co-localized in the nucleus. Bars: 10 μm. **b** Transcription activation ability of MabHLH1. Yeasts transformed with the positive control (pGBKT7-53 + pGADT7-T) and negative control (pGBKT7), and pGBKT7/ MabHLH1 vectors were each cultivated in SD/-Trp media, SD/-Trp media with 40 mg mL^− 1^ X-α-gal, and SD/-Trp media plus 40 mg mL^− 1^ X-α-gal and 200 ng mL^− 1^ AbA. The positive control and pGBKT7/MabHLH1 exhibited blue yeast plaques, while the negative control did not grow in SD/-Trp media plus 40 mg mL^− 1^ X-α-gal and 200 ng/mL AbA. (DOCX 280 kb)

Additional file 4: **Figure S4.** Anthocyanin composition analysis of the leaves and corollas in the other two lines of control, OE-*MaAN2*, and OE-*MaMybA* tobaccos. High-performance liquid chromatography analysis of anthocyanin extract. According to the chromatographic peak in the sequence, the standards are delphinidin 3-rutinoside, cyanidin 3-rutinoside, and pelargonidin 3-rutinoside (from left to right). (DOCX 228 kb)

Additional file 5: **Figure S5.** Mass spectrum and structure patterns of cyanidin-3-rutinoside. (DOCX 153 kb)

Additional file 6: **Figure S6.** Histological localization of anthocyanins in leaf tissues of the control, OE-*MaAN2*, and OE-*MaMybA* tobaccos. The genotypes are the control (**a-d**), OE-*MaAN2* (**e-h**), and OE-*MaMybA* (**i-l**); abaxial epidermis (**a**, **e**, and **i**), trichomes (**b**, **f**, and **j**), cross-sections through leaves (**c**, **g**, and **k**), and leaf veins (**d**, **h**, and **l**). ep, epidermal; hy, hypodermal; tr, trichome; pa, parenchyma; pp., palisade parenchyma; st, stomata; sp., spongy parenchyma; va, vascular bundle. Bars: 100 μm. (DOCX 550 kb)

Additional file 7: **Figure S7.** Scanning electron microscopy images of leaf and corolla adaxial surface cells in different tobacco genotypes. The leaf (**a**-**c**) and corolla (**d-f**) adaxial surface cells are shown for the control (**a**, **d**), OE-*MaAN2* (**b**, **e**), and OE-*MaMybA* (**c**, **f**) tobacco. (DOCX 145 kb)

Additional file 8: **Table S1.** Primers used in this study. (DOCX 22 kb)

## Data Availability

All data generated or analyzed during this study are included in this published article and its supplementary information files.

## Abbreviations

AbA: Aureobasidin A
ANS: Anthocyanidin synthase
bHLH: Basic helix-loop-helix
BiFC: Bimolecular fluorescence complementation
CHI: Chalcone isomerase
CHS: Chalcone synthase
Cy: Cyanidin
Cy3R: Cyanidin 3-rutinoside
DFR: Dihydroflavonol 4-reductase
Dp: Delphinidin
Dp3R: Delphinidin 3-rutinoside
EBG: Early biosynthetic gene
F3’H: Flavonoid 3′-hydroxylase
F3′5’H: Flavonoid 3’5’-hydroxylase
F3H: Flavanone 3-hydroxylase
GFP: Green fluorescent protein
HPLC: High-performance liquid chromatography
LBG: Late biosynthetic gene
LUC: Firefly luciferase
m/z: Mass-to-charge ratio
MS: Mass spectrometry
MS/MS: tandem mass spectrometry
OE: Overexpressing
ORF: Open reading frame
qRT-PCR: Quantitative real-time polymerase chain reaction
REN: *Renilla* luciferase
RT-PCR: Reverse transcription-polymerase chain reaction
TF: Transcription factor
TOF: Time-of-flight
UFGT: Uridine diphosphate-sugar: flavonoid glycosyltransferases
UPLC: Ultra-performance liquid chromatography
WDR: WD-repeat
YFP: Yellow fluorescent protein
