# Supplementary figures and images for: A novel R2R3-MYB from grape hyacinth, MaMybA, which is different from MaAN2, confers intense and magenta anthocyanin pigmentation in tobacco
Source: BMC Plant Biol. 2019 Sep 9;19:390. doi: 10.1186/s12870-019-1999-0 (PMC6734322; doi:10.1186/s12870-019-1999-0)

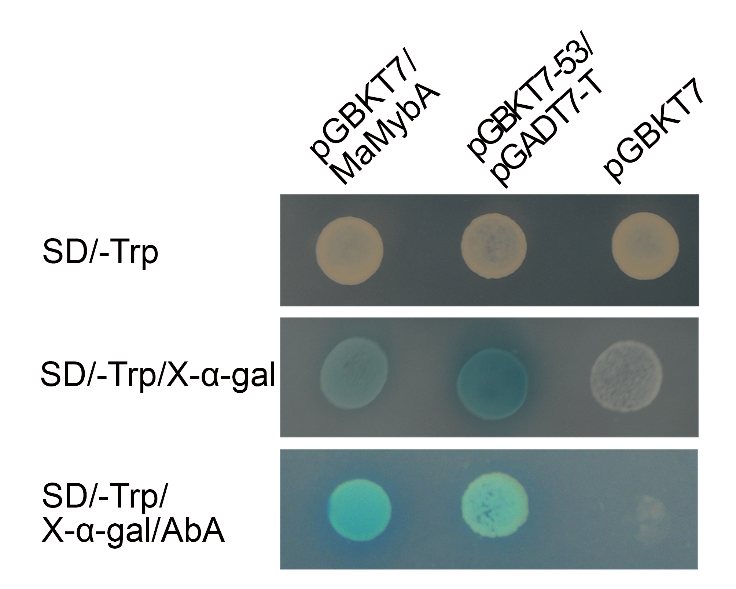


**Addition file 1_ Figure S1**

Supplement: Supplementary file 1 — Figure S1. Transcription activation ability of MaMybA. Yeasts transformed with the pGBKT7/MaMybA vector (positive control (pGBKT7-53 + pGADT7-T) and negative control (pGBKT7)), were each cultured in SD/-Trp media, SD/-Trp media with 40 mg mL− 1 X-α-gal, and SD/-Trp media added to 40 mg mL− 1 X-α-gal and 200 ng mL− 1 AbA. (DOCX 273 kb) [file 12870_2019_1999_MOESM1_ESM.docx]

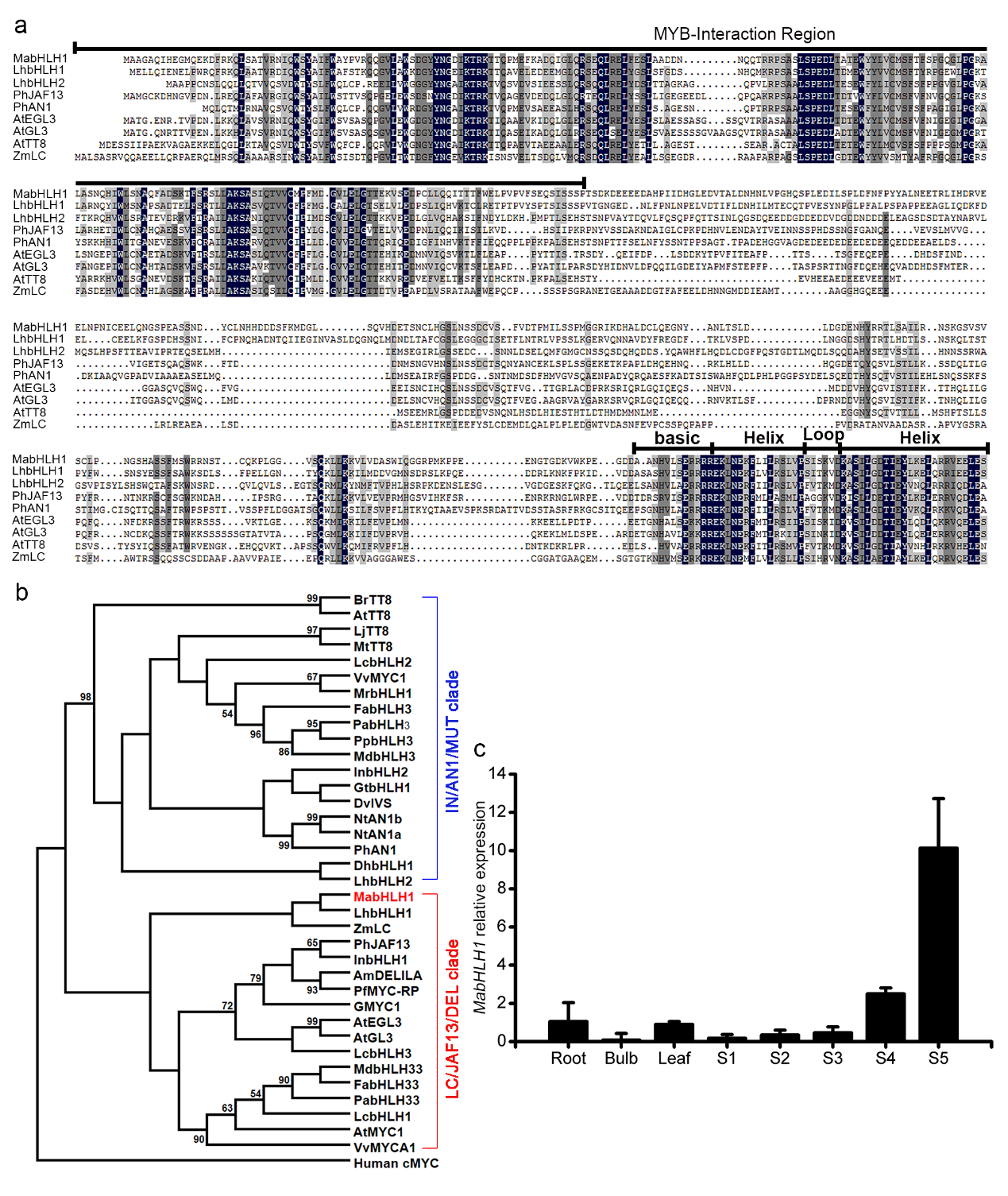


**Addition file 2_ Figure S2**

Supplement: Supplementary file 2 — Figure S2. Alignment and phylogenetic analysis of the deduced amino acid sequence of MabHLH1 and other basic Helix-Loop-Helix (bHLH) proteins associated with flavonoid biosynthesis from other plants. a Sequence alignment of partial amino acids deduced from MabHLH1 and other bHLH proteins from other plant species. The MYB-interaction and bHLH DNA binding regions are indicated above the alignment by bold black lines. b Phylogenetic analysis of entire amino acid sequences deduced from MabHLH1 and other bHLH proteins from other plants. The maximum-likelihood phylogenetic tree was generated using MEGA 6.0 software. Numbers next to the nodes indicate the bootstrap values from 1000 replications. The bHLH protein sequences of different plant species were retrieved from GenBank database and their GenBank accession numbers are as followings: Arabidopsis thaliana: AtTT8 (NP_1927202), AtEGL3 (NP_0011853021), AtGL3 (NP_0013327061), and AtMYC1 (AEE818881); Petunia hybrida: PhJAF13 (AAC394551) and PhAN1 (AAG259281); Nicotiana tabacum: NtAN1a (AEE992571) and NtAN1b (AEE992581); Antirrhinum majus: AmDELILA (AAA326631); Gerbera hybrida: GMYC1 (CAA076151); Vitis vinifera: VvMYC1 (ACC686851) and VvMYCA1 (ABM923323); Malus domestica: MdbHLH3 (ADL365971) and MdbHLH33 (ABB844741); Myrica rubra: MrbHLH1 (AGO583721); Perilla frutescens: PfMYC-RP (BAA75513); Brassica rapa: BrTT8 (AEA032811); Medicago truncatula: MtTT8 (AKN796061); Lotus japonicus: LjTT8 (BAH288811); Dahlia pinnata: DvIVS (BAJ335151); Ipomoea nil: InbHLH1 (BAE943931) and InbHLH2 (BAE943941); Fragaria×ananassa: FabHLH3 (AFL024631) and FabHLH33 (AFL024651); Prunus persica: PpbHLH3 (AIE575081); Prunus avium: PabHLH3 (AJB284811) and PabHLH33 (AJB284841); Litchi chinensis: LcbHLH3 (APP941241), LcbHLH1 (APP941221), and LcbHLH2 (APP941231); Lilium hybrid: LhbHLH1 (BAE200571) and LhbHLH2 (BAE200581); Zea mays: ZmLC (NP_0011053391); Dendrobium hybrid: DhbHLH1 (AQS798531); Gentiana triflora: GtbHLH1 (BAH033871). c Expression profile of [file 12870_2019_1999_MOESM2_ESM.docx]

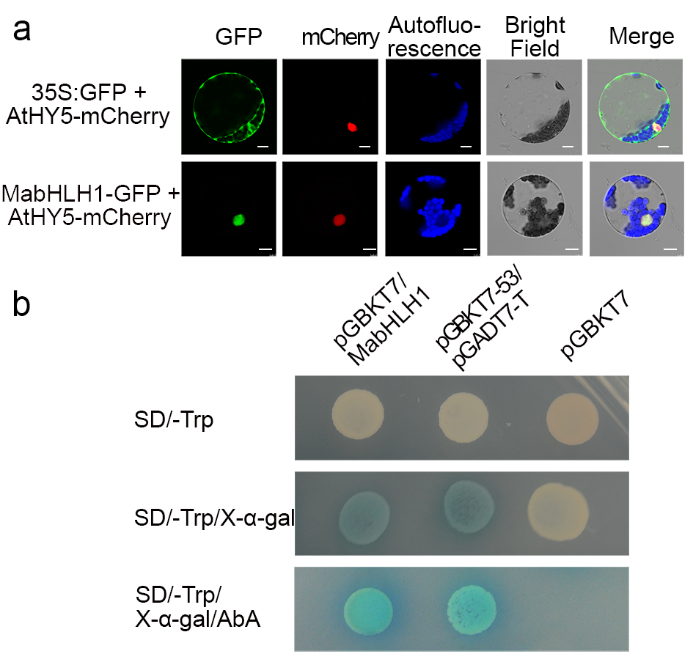


**Addition file 3_ Figure S3**

Supplement: Supplementary file 3 — Figure S3. Subcellular localization and transcription activation ability of MabHLH1. a Subcellular localization of MabHLH1. The transient co-expression of MabHLH1-GFP with AtHY5-mCherry in Arabidopsis thaliana mesophyll protoplasts showed that MabHLH1 and AtHY5 were co-localized in the nucleus. Bars: 10 μm. b Transcription activation ability of MabHLH1. Yeasts transformed with the positive control (pGBKT7-53 + pGADT7-T) and negative control (pGBKT7), and pGBKT7/ MabHLH1 vectors were each cultivated in SD/-Trp media, SD/-Trp media with 40 mg mL− 1 X-α-gal, and SD/-Trp media plus 40 mg mL− 1 X-α-gal and 200 ng mL− 1 AbA. The positive control and pGBKT7/MabHLH1 exhibited blue yeast plaques, while the negative control did not grow in SD/-Trp media plus 40 mg mL− 1 X-α-gal and 200 ng/mL AbA. (DOCX 280 kb) [file 12870_2019_1999_MOESM3_ESM.docx]

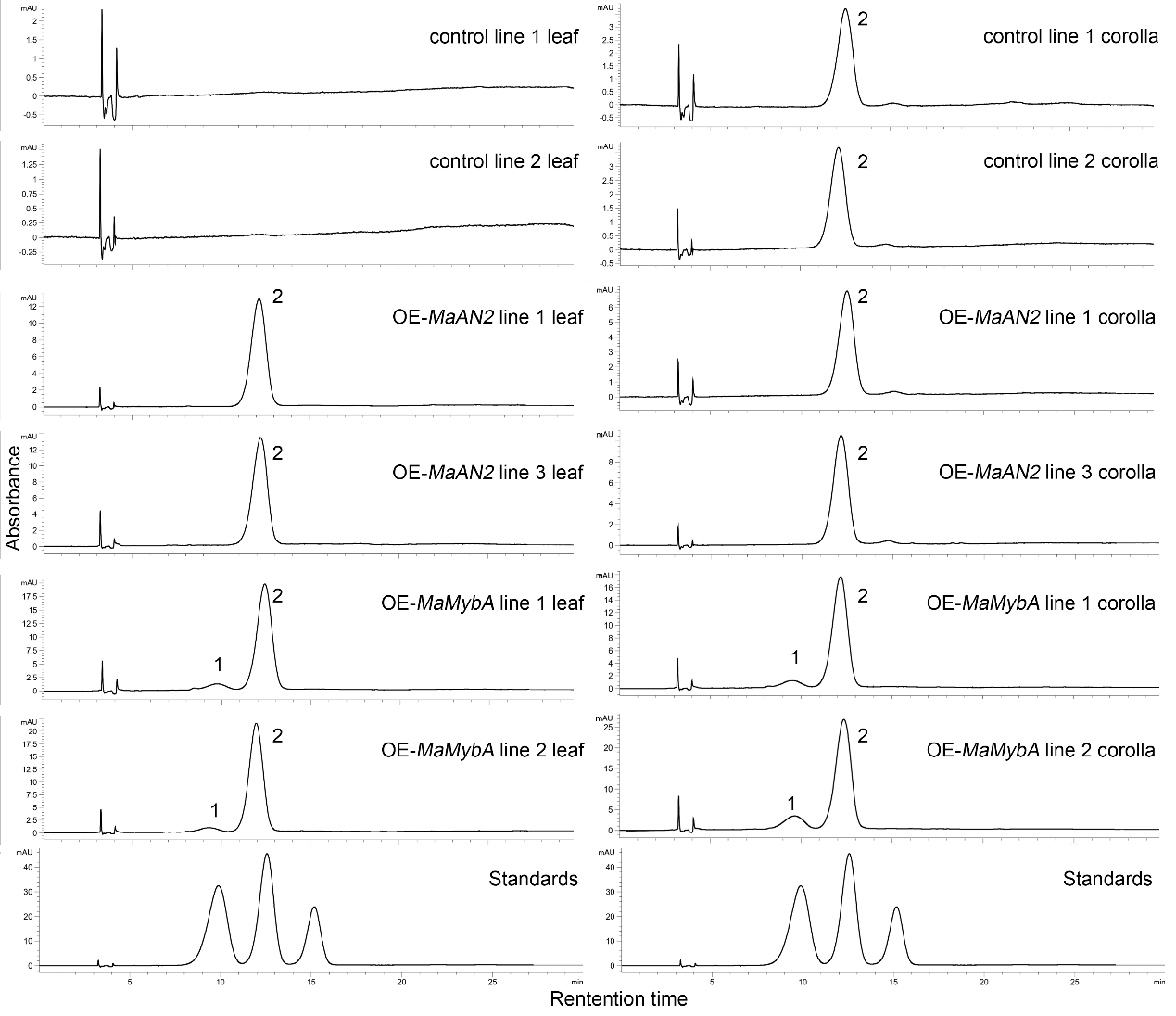


**Addition file 4_ Figure S4**

Supplement: Supplementary file 4 — Figure S4. Anthocyanin composition analysis of the leaves and corollas in the other two lines of control, OE-MaAN2, and OE-MaMybA tobaccos. High-performance liquid chromatography analysis of anthocyanin extract. According to the chromatographic peak in the sequence, the standards are delphinidin 3-rutinoside, cyanidin 3-rutinoside, and pelargonidin 3-rutinoside (from left to right). (DOCX 228 kb) [file 12870_2019_1999_MOESM4_ESM.docx]

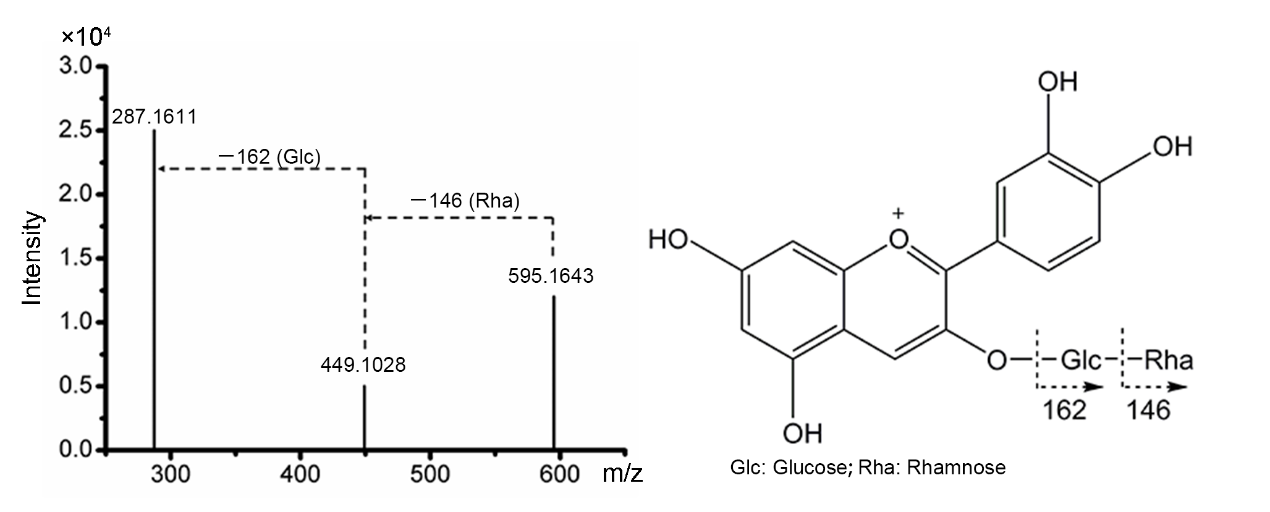


**Addition file 5_ Figure S5**

Supplement: Supplementary file 5 — Figure S5. Mass spectrum and structure patterns of cyanidin-3-rutinoside. (DOCX 153 kb) [file 12870_2019_1999_MOESM5_ESM.docx]

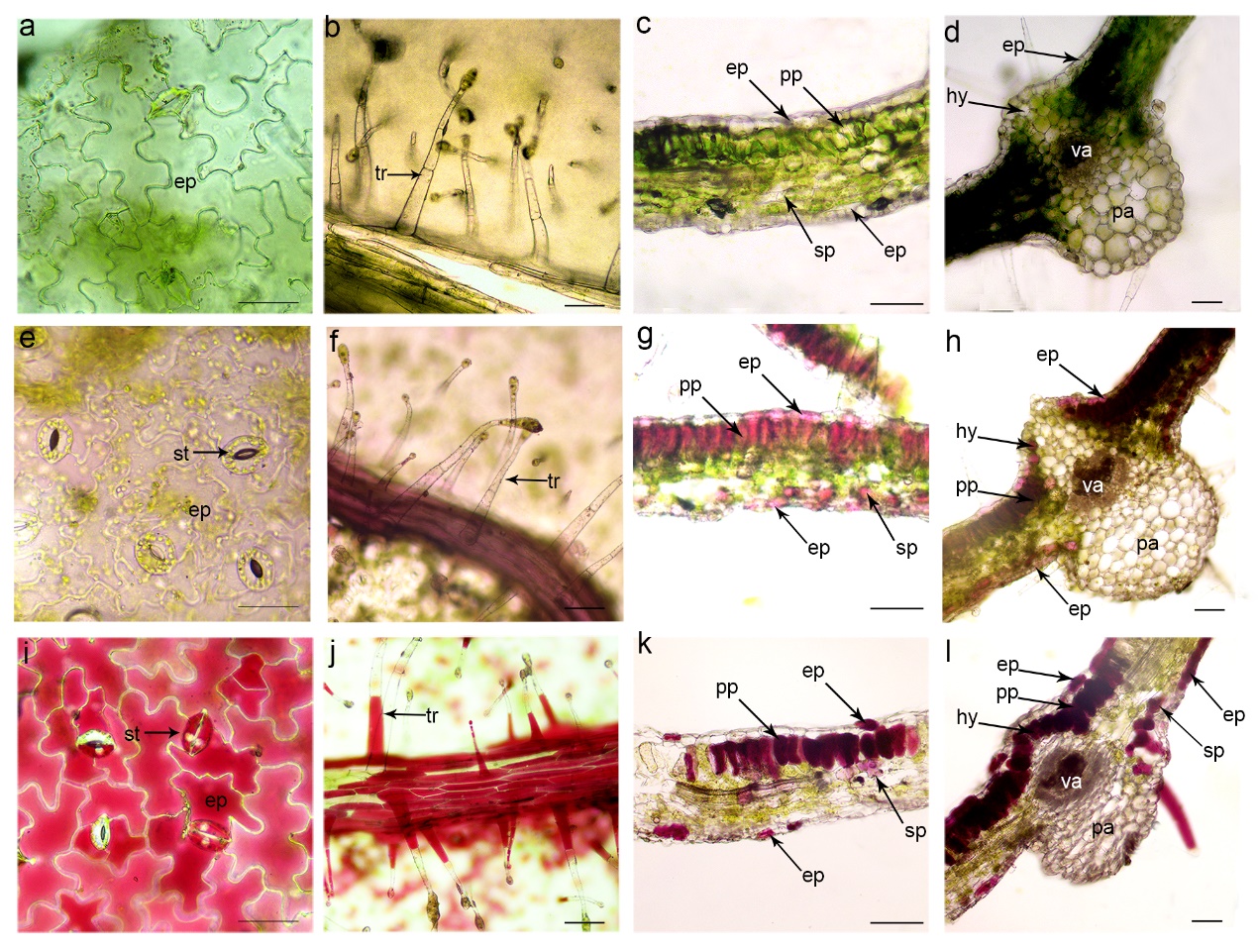


**Addition file 6_ Figure S6**

Supplement: Supplementary file 6 — Figure S6. Histological localization of anthocyanins in leaf tissues of the control, OE-MaAN2, and OE-MaMybA tobaccos. The genotypes are the control (a-d), OE-MaAN2 (e-h), and OE-MaMybA (i-l); abaxial epidermis (a, e, and i), trichomes (b, f, and j), cross-sections through leaves (c, g, and k), and leaf veins (d, h, and l). ep, epidermal; hy, hypodermal; tr, trichome; pa, parenchyma; pp., palisade parenchyma; st, stomata; sp., spongy parenchyma; va, vascular bundle. Bars: 100 μm. (DOCX 550 kb) [file 12870_2019_1999_MOESM6_ESM.docx]

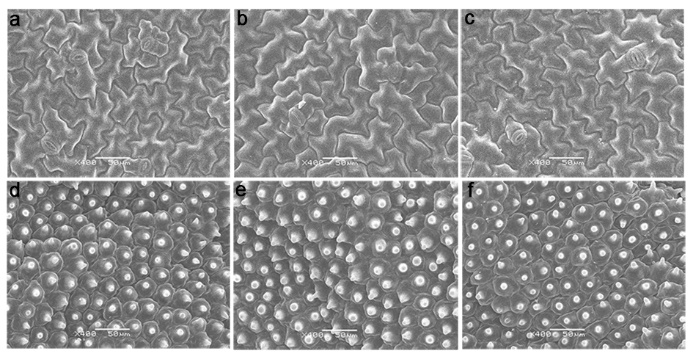


**Addition file 7_ Figure S7**

Supplement: Supplementary file 7 — Figure S7. Scanning electron microscopy images of leaf and corolla adaxial surface cells in different tobacco genotypes. The leaf (a-c) and corolla (d-f) adaxial surface cells are shown for the control (a, d), OE-MaAN2 (b, e), and OE-MaMybA (c, f) tobacco. (DOCX 145 kb) [file 12870_2019_1999_MOESM7_ESM.docx]
