# Supplementary material for: A novel R2R3-MYB from grape hyacinth, MaMybA, which is different from MaAN2, confers intense and magenta anthocyanin pigmentation in tobacco
Source: BMC Plant Biol. 2019 Sep 9;19:390. doi: 10.1186/s12870-019-1999-0 (PMC6734322; doi:10.1186/s12870-019-1999-0)
Supplement: Supplementary file 8 — Table S1. Primers used in this study. (DOCX 22 kb) [file 12870_2019_1999_MOESM8_ESM.docx]

Table S1 Primers used in this study

| Purpose | Primer name | Primer sequence (5′→3′) |
| --- | --- | --- |
| Cloning genes | MaMybA-5′RACE | CATCCTCCTCCTCCTCCGTCGCTGT |
|  | MaMybA-3′RACE | CTTGGGCAACAGGTGGTCGCTAATC |
|  | MaMybA-full length cDNA-F | ATGGAACCCAAGCTCAAGTCATC |
|  | MaMybA-full length cDNA-R | TCATAACAAGTTATGGGCATGGAGC |
|  | MabHLH1-full length cDNA-F | ATGGCAGCGGGGGCTCAAATC |
|  | MabHLH1-full length cDNA-R | TCAGCGTTTGCTGGCAACTCTGTG |
| Subcellular localization | MaMybA-pC2300GFP-F-*Bam*HⅠ | CGAGCTCGGTACCCGGGGATCCATGGAACCCAAGCTCAAGTCATC |
|  | MaMybA-pC2300GFP-R-*Sal*Ⅰ | CTTGCTCACCATGGTGTCGACTAACAAGTTATGGGCATGGAGCAG |
|  | MabHLH1-pC2300GFP-F-*Bam*HⅠ | CGAGCTCGGTACCCGGGGATCCATGGCAGCGGGGGCTCAAATC |
|  | MabHLH1-pC2300GFP-R-*Sal*Ⅰ | CTTGCTCACCATGGTGTCGACGCGTTTGCTGGCAACTCTGTGA |
|  | AtHY5-pBI221Cherry-F-*Sma*Ⅰ | TGTACAAGGAATTCCCGGGATGCAGGAACAAGCGACTAGCT |
|  | AtHY5- pBI221mCherry-R-*Sac*Ⅰ | GATCGGGGAAATTCGAGCTCAAGGCTTGCATCAGCATTAGAACC |
| Transcription activation assay | MaMybA-pGBKT7-F-*Nde*Ⅰ | CAGAGGAGGACCTGCATATGGAACCCAAGCTCAAGTCATC |
|  | MaMybA-pGBKT7-R-*Bam*HⅠ | CGCTGCAGGTCGACGGATCCTCATAACAAGTTATGGGCATGGAGC |
|  | MabHLH1-pGBKT7-F-*Nde*Ⅰ | CAGAGGAGGACCTGCATATGGCAGCGGGGGCTCAAATC |
|  | MabHLH1-pGBKT7-R-*Bam*HⅠ | CGCTGCAGGTCGACGGATCCTCAGCGTTTGCTGGCAACTCTGTG |
| qRT-PCR assay | MaMybA-F | TCCAGAGGTGTCGAAAGA |
|  | MaMybA-R | TGTGTAGGCGCATGATAAG |
|  | MabHLH1-F | GCAATGCTCAGTTTGCAGATAG |
|  | MabHLH1-R | CTCTAGGACACCATCCATGAAG |
|  | MaAN2-F | CATGAACGGCAGACGAATCT |
|  | MaAN2-R | GAAATTAGCATTCAAGCCATCCC |
|  | MaActin-F | AACATTCAGAAAGAGTCCACCC |
|  | MaActin-R | GCTTACCAGCAAAGATCAACCG |
|  | NtCHS-F | TGACACCCACTTGGATAGTTTAG |
|  | NtCHS-R | CGACCTCTGGAATTGGATCAG |
|  | NtCHI-F | CTTTTCTCGCCGCTAAATG |
|  | NtCHI-R | TTTCTGCCACCTTCTCTG |
|  | NtF3H-F | CAAGGCATGTGTGGATATGG |
|  | NtF3H-R | TGTGTCGTTTCAGTCCAAGG |
|  | NtF3′H-F | AGGCTCAACACTTCTCGT |
|  | NtF3′H-R | CATCAACTTTGGGCTTCT |
|  | NtF3′5′H-F | CGCACTACCATACTTAGGAGCCAT |
|  | NtF3′5′H-R | CAGCATCAGGAGTAGAAGCAACAG |
|  | NtDFR-F | AACCAACAGTCAGGGGAATG |
|  | NtDFR-R | TTGGACATCGACAGTTCCAG |
|  | NtANS-F | TGGCGTTGAAGCTCATACTG |
|  | NtANS-R | GGAATTAGGCACACACTTTGC |
|  | NtUFGT-F | GAGTGCATTGGATGCCTTTT |
|  | NtUFGT-R | CCAGCTCCATTAGGTCCTTG |
|  | NtAN2-F | GAAGAAAGGTGCATGGACTG |
|  | NtAN2-R | TCTGCAGCTCTTTCTGCATC |
|  | NtAn1a-F | ACCATTCTCGAACACCGAAG |
|  | NtAn1a-R | TGCTAGGGCACAATGTGAAG |
|  | NtAn1b-F | CTTGAACACTTCTCAAACCGA |
|  | NtAn1b-R | TGCTAGGGCACAATGTGAAG |
|  | NtTubA1-F | CTCCTATGCTCCTGTCATTTC |
|  | NtTubA1-R | GGCGAGGATCACACTTAAC |
| BiFC assays | MaAN2-pSPYNE-F-*Bam*HⅠ | CCAGGCCTACTAGTGGATCCATGGGAGCCCCTTCAACCTC |
|  | MaAN2-pSPYNE-R-*Sal*Ⅰ | GCGGTACCCTCGAGGTCGACCTAAATAGAATCGAAATCAAACCAAAGGTTG |
|  | MabHLH1-pSPYNE-F-*Bam*HⅠ | CCAGGCCTACTAGTGGATCCATGGCAGCGGGGGCTCAAATC |
|  | MabHLH1-pSPYNE-R-*Sal*Ⅰ | GCGGTACCCTCGAGGTCGACTCAGCGTTTGCTGGCAACTCTGTG |
|  | MaMybA-pSPYCE-F-*Bam*HⅠ | GGCGCGCCACTAGTGGATCCATGGAACCCAAGCTCAAGTCATC |
|  | MaMybA-pSPYCE-R-*Sal*Ⅰ | GCGGTACCCTCGAGGTCGACTCATAACAAGTTATGGGCATGGAGC |
|  | MabHLH1-pSPYCE-F-*Bam*HⅠ | GGCGCGCCACTAGTGGATCCATGGCAGCGGGGGCTCAAATC |
|  | MabHLH1-pSPYCE-R-*Sal*Ⅰ | GCGGTACCCTCGAGGTCGACGCGTTTGCTGGCAACTCTGTGA |
| Dual luciferase assay | MaMybA-pGreeni62-SK-F-*Eco*RⅠ | CCCCCGGGCTGCAGGAATTCATGGAACCCAAGCTCAAGTCATC |
|  | MaMybA-pGreenⅡ62-SK-R-*Kpn*Ⅰ | GATTTCAGCGAATTGGTACCTCATAACAAGTTATGGGCATGGAGC |
|  | MaAN2-pGreenⅡ62-SK-F-*Eco*RⅠ | CCCCCGGGCTGCAGGAATTCATGGGAGCCCCTTCAACCTC |
|  | MaAN2-pGreenⅡ62-SK-R-*Kpn*Ⅰ | GATTTCAGCGAATTGGTACCCTAAATAGAATCGAAATCAAACCAAAGGTTG |
|  | MabHLH1-pGreenⅡ62-SK-F-*Eco*RⅠ | AGGAATTCGATATCAAGCTTATGGCAGCGGGGGCTCAAATC |
|  | MabHLH1-pGreenⅡ62-SK-R-KpnⅠ | GATTTCAGCGAATTGGTACCTCAGCGTTTGCTGGCAACTCTGTG |
|  | pMaCHS-pGreenⅡ0800-LUC-F-*Sal*Ⅰ | GGCCCCCCCTCGAGGTCGACGTGAAGAAACAGAGAGGGCCATGG |
|  | pMaCHS-pGreenⅡ0800-LUC-R-*Bam*HⅠ | GCTCTAGAACTAGTGGATCCGAGATAAAAGGATGGGAAGAATGACAAATATAATG |
|  | pMaDFR-pGreenⅡ0800-LUC-F-*Sal*Ⅰ | GGCCCCCCCTCGAGGTCGACGTGGAGTAGCATAGGAACGAGAG |
|  | pMaDFR-pGreenⅡ0800-LUC-R-*Bam*HⅠ | GCTCTAGAACTAGTGGATCCCTTGTTTGTGTGATTGTGTTGGGAG |
|  | pAtDFR-pGreenⅡ0800-LUC-F-*Sal*Ⅰ | GGCCCCCCCTCGAGGTCGACTCTGACGTCTTACGATACAACAAATTG |
|  | pAtDFR-pGreenⅡ0800-LUC-R-*Bam*HⅠ | GCTCTAGAACTAGTGGATCCTTTTGTGGTTATATGATAGATTGTGCTTTG |
| Tobacco transformation | MaMybA-pC1304-F-NcoⅠ | CGGGGGACTCTTGACCATGGGAGCCCCTTCAACCTC |
|  | MaMybA-pC1304-R-*Bgl*Ⅱ | CTCCTTTACTAGTCAGATCTTCAATAGAATCGAAATCAAACCAAAGGTTGAAAT |
|  | MaAN2-pC1304-F-*Nco*Ⅰ | CGGGGGACTCTTGACCATGGGAGCCCCTTCAACCTC |
|  | MaAN2-pC1304-R-BglⅡ | CTCCTTTACTAGTCAGATCTAATAGAATCGAAATCAAACCAAAGGTTGAAAT |
